# Enzymatic properties and subtle differences in the substrate specificity of phylogenetically distinct invertebrate *N*-glycan processing hexosaminidases

**DOI:** 10.1093/glycob/cwu132

**Published:** 2014-12-08

**Authors:** Martin Dragosits, Shi Yan, Ebrahim Razzazi-Fazeli, Iain B H Wilson, Dubravko Rendic

**Affiliations:** 2 Department of Chemistry, University of Natural Resources and Life Sciences, Vienna; 3 VetCore Facility for Research, University of Veterinary Medicine, Vienna, Austria

**Keywords:** fused lobes, hexosaminidase, insect, invertebrate, *N*-glycans

## Abstract

Fused lobes (FDL) hexosaminidases are the most recently genetically defined glycosidases involved in the biosynthesis of *N*-glycans in invertebrates, and their narrow specificity is essential for the generation of paucimannosidic *N*-glycans in insects. In this study, we explored the potential of FDL hexosaminidases in the utilization of different artificial and natural substrates, both as purified, native compounds or generated in vitro using various relevant glycosyltransferases. In addition to the already-known FDL enzyme from *Drosophila melanogaster*, we now have identified and characterized the *Apis mellifera* FDL homolog. The enzymatic properties of the soluble forms of the affinity-purified insect FDL enzymes, expressed in both yeast and insect cells, were compared with those of the phylogenetically distinct recombinant *Caenorhabditis elegans* FDL-like enzymes and the *N*-acetylgalactosamine (GalNAc)-specific *Caenorhabditis* hexosaminidase HEX-4. In tests with a range of substrates, including natural *N*-glycans, we show that the invertebrate FDL(-like) enzymes are highly specific for *N*-acetylglucosamine attached to the α1,3-mannose, but under extreme conditions also remove other terminal GalNAc and *N*-acetylglucosamine residues. Recombinant FDL also proved useful in the analysis of complex mixtures of *N*-glycans originating from wild-type and mutant *Caenorhabditis* strains, thereby aiding isomeric definition of paucimannosidic and hybrid *N*-glycans in this organism. Furthermore, differences in activity and specificity were shown for two site-directed mutants of *Drosophila* FDL, compatible with the high structural similarity of chitinolytic and *N*-glycan degrading exohexosaminidases in insects. Our studies are another indication for the variety of structural and function aspects in the GH20 hexosaminidase family important for both catabolism and biosynthesis of glycoconjugates in eukaryotes.

## Introduction

One of the most obvious features of invertebrate *N*-glycomes is the presence of paucimannosidic structures, i.e. *N*-glycan structures consisting of just (un)modified tri- or bimannosylchitobiosyl cores. The enzymes essential for the final steps of the biosynthesis of these structures are hexosaminidases which remove the non-reducing *N*-acetylglucosamine (GlcNAc) initially added to the *N*-glycan by *N*-acetylglucosaminyltransferase I (GnTI). The presence of these hexosaminidases was initially detected in the protein extracts of several insect cell lines (Altmann, Schwihla, et al. 1995). A decade later, we identified and characterized the sequence encoding for the relevant *Drosophila melanogaster* enzyme (Léonard et al. 2006), named fused lobes (FDL) hexosaminidase after the mushroom body FDL phenotype observed in the corresponding *D. melanogaster* mutant (Boquet et al. 2000).

Based on its sequence, FDL is classified as a member of the CAZy family GH20. The hallmark of the FDL hexosaminidases is their apparently strict preference for the non-reducing, terminal GlcNAc residue on the α1,3-arm of an *N*-glycan structure and deletion of the gene results in a highly altered *N*-glycome. All other known β-hexosaminidases examined to date are either able to remove both α1,3- and α1,6-arm terminal GlcNAc residues from an *N*-glycan [e.g. mammalian lysosomal enzymes (Hepbildikler et al. 2002), the *A. thaliana* HEX-1 and HEX-2 (Gutternigg et al. 2007)] or are unable to remove any of the terminal GlcNAc residues from an *N-*glycan [non-FDL *Drosophila* hexosaminidases (Léonard et al. 2006), the *A. thaliana* HEX-3 and *Caenorhabditis* HEX-1, HEX-4 and HEX-5 (Gutternigg et al. 2007)]. This narrow specificity of the FDL hexosaminidases has potential to be utilized in the structural determination of the biantennary *N-*glycan structures by specifically removing terminal α1,3-arm *N*-acetylglucosamine. Currently, such structural evaluations require non-trivial high performance liquid chromatography (HPLC) runs, acetolysis or mass-spectrometric (MS^n^) analyses of permethylated structures.

Since the characterization of the *Drosophila* hexosaminidases, intracellular localization studies indicated that FDL is present in the secretory pathway (Léonard et al. 2006) and homologs of other insect FDL hexosaminidases were identified and characterized (Geisler et al. 2008; Geisler and Jarvis 2010; Nomura et al. 2010; Pasini et al. 2011; Huo et al. 2013). Jarvis and his coworkers indicated that the *Drosophila* and *Spodoptera frugiperda* FDL hexosaminidases are resident Golgi enzymes (Geisler and Jarvis 2012). The general conclusion is that the action of FDL hexosaminidases constitutes a regular and deliberate event during glycoprotein maturation occurring within the insect protein secretory pathway. These findings contribute critical information toward on-going efforts in changing the insect glycosylation machinery to resemble the mammalian one (Hollister et al. 2002; Watanabe et al. 2002; Aumiller et al. 2003, 2012; Tomiya et al. 2003); indeed, insect-derived cells are an established expression system for the production of recombinant (glyco)proteins (e.g. antibodies; Palmberger et al. 2011), including therapeutical ones (Cox 2012). Apart from insects, other invertebrates also express hexosaminidases with FDL-like activity: we could show that *Caenorhabditis elegans* possesses two differentially expressed hexosaminidases (HEX-2 and HEX-3) which also remove the GlcNAc residue added by the GnTI, although these enzymes are phylogenetically distant from the insect FDL enzymes (Gutternigg et al. 2007). A follow-up analysis of the double mutant of the *Caenorhabditis* HEX-2 and HEX-3 has clearly demonstrated that both HEX-2 and HEX-3 contribute toward the maturation of *N-*glycans in this organism (Yan et al. 2012).

The FDL enzymes differ from other β-hexosaminidases found in invertebrates, plants and mammals. Although mammals possess various β-hexosaminidases, which include catabolic lysosomal hexosaminidases (Hex A and Hex B; Korneluk et al. 1986), a nucleocytoplasmic hexosaminidase (Hex D; Gutternigg et al. 2009) and the hexosaminidase involved in O-GlcNAc turnover (EC 3.2.1.169, CAZy family GH84; Gao et al. 2001; Wells et al. 2002), these are not present in the Golgi apparatus. On the other hand, even though the major, mature plant *N-*glycan structures resemble those found in insects [e.g. Man_3_GlcNAc_2_FucXyl *N-*glycan structure found in flowers of *Arabidopsis thaliana* and *Nicotiana benthamiana* (Rendic et al. 2007) in comparison with the Man_3_GlcNAc_2_Fuc *N-*glycan structure found in *Drosophila* (Fabini et al. 2001; Rendic et al. 2006)], the biosynthesis pathway of these structures in plants does not appear to involve FDL-like enzymes. Indeed, the hexosaminidases described in plants to date are predicted to be localized in the vacuole/plasma membrane (Vitale and Chrispeels 1984; Gutternigg et al. 2007; Liebminger et al. 2011) or to be involved in chitin degradation (Gutternigg et al. 2007). Furthermore, secreted plant glycoproteins (such as laccase) often contain extended structures with “Lewis a” epitopes (Fitchette-Laine et al. 1997). Insects, apart from the FDL enzymes, also express other hexosaminidases. In *Drosophila*, the hexosaminidases Hexo1 and Hexo2 were shown to act on chitin-derived substrates and actually are unable to remove non-reducing terminal GlcNAc residues from a typical *N-*glycan structure (Léonard et al. 2006). More recently, it was shown that the Hexo1 is responsible for removal of one of the two residual GlcNAc residues from the degraded *N-*glycan during biosynthesis of the *Drosophila* rhodopsin 1 (Rosenbaum et al. 2014). In contrast, both described non-FDL hexosaminidases from *S. frugiperda* are also able to process typical biantennary *N-*glycan structures (Tomiya et al. 2006; Geisler et al. 2008).

In this study, we have investigated the suitability of FDL hexosaminidases in the analysis of *N-*glycans. For the first time, purified forms of the recombinant FDL(-like) enzymes from *Caenorhabditis* and *Drosophila* were used to measure and compare their activity toward various *p*-nitrophenyl-monosaccharides. Also, as an important extension of our previous work, we were able to identify and characterize the *Apis mellifera* homolog of the FDL hexosaminidase. Differently modified *N*-glycopeptides terminating with either GlcNAc or *N*-acetylgalactosamine (GalNAc) residues were prepared and tested as substrates for all enzymes used in this study. A comparison of the *Drosophila*, *Caenorhabditis* and *Apis* FDL(-like) hexosaminidases is provided. Furthermore, analysis of site-directed mutants of *Drosophila* FDL indicates a high structural similarity of chitin- and *N-*glycan degrading hexosaminidases in insects. Finally, we have clarified the position of the terminal, non-reducing β-GlcNAc on a number of *N-*glycans carrying this residue by utilizing the purified *Drosophila* FDL hexosaminidase for the structural analysis of the complex mixtures of *N-*glycans from wild-type and mutant *Caenorhabditis* strains.

## Results

### Production of recombinant FDL enzymes

**Fig. 1.**
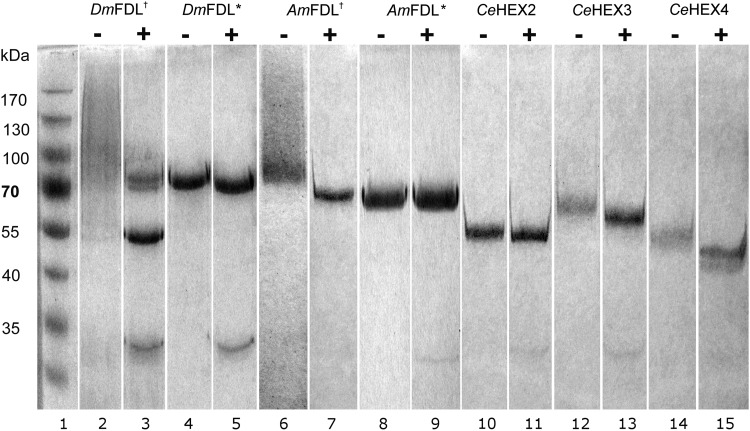
SDS–PAGE analysis of purified, recombinant hexosaminidases. Purified recombinant enzymes were either incubated with water (−) or *N*-glycosidase F (+) and subjected to SDS–PAGE and staining with Coomassie Brilliant Blue. Dagger indicates the enzyme produced in *P. pastoris* X-33 and asterisk indicates the enzyme produced in Hi5 insect cells. Band at approximately 30 kDa in (+) lanes corresponds to the *N*-glycosidase F protein. All *C. elegans* enzymes were produced in *P. pastoris* X-33.

In an effort to produce the recombinant FDL enzyme of high purity suitable for a thorough study of its properties, we have analyzed the expression of various FDL(-like) enzymes using two different expression systems. Prompted by the success in the previous study (Léonard et al. 2006), we have initially expressed the *D. melanogaster* FDL in *Pichia pastoris*. The activity of the recombinant protein carrying the C-terminal HIS-tag could not be detected, whereas the purification of the protein carrying the *N*-terminal HIS-tag yielded moderate amounts (88 mU mL^−1^) of the partly degraded recombinant protein (Figure 1, lanes 2 and 3). In an effort to increase the yield and quality of the recombinant product, common parameters (culture temperature, medium type) were varied, followed by the bioreactor pilot expression of the recombinant enzyme (Supplementary data, Figure S1). In addition, we used a codon-optimized version of the open reading frame (ORF), which finally lead to a minor increase of 25% in yield using optimal medium (YP) and optimal temperature (25°C) (Table I and Supplementary data, Figure S2). Therefore, we decided to also express the *Drosophila* FDL using the Baculovirus expression system in insect cells. The affinity-purified recombinant enzyme, when analyzed by sodium dodecyl sulphate –polyacrylamide gel electrophoresis (SDS–PAGE), migrated as single band of an expected size (Figure 1, lanes 4 and 5). Moreover, the insect expression system yielded higher amounts of the recombinant *Drosophila* FDL (Table I).

**Table I.** Activity of enzymes recombinantly expressed in two expression systems

Protein	*Pichia pastoris* (mU mL^−1^)	Hi5 insect cells (mU mL^−1^)
DmFDL	88	n/d
DmFDL^a^	110	2500
AmFDL	150	250
CeHEX-2	2100	n/d
CeHEX-3	240	n/d
CeHEX-4	870	n/d

Units were calculated based on the pNP-β-GlcNAc for insect and on pNP-β-GalNAc for the *Caenorhabditis* enzymes. Assays were performed at the pH optimum of the respective enzymes. DmFDL assays were performed at 30°C, whereas all other enzymes were tested at 37°C. n/d, not determined.

^a^*P. pastoris* codon-optimized protein.

In parallel, we also expressed and purified *C. elegans* HEX-2 and HEX-3, enzymes previously described as enzymes displaying FDL-like activity in our laboratory (Gutternigg et al. 2007). For comparison purposes, *Caenorhabditis* HEX-4, an enzyme previously described to specifically remove terminal β-GalNAc residues (Gutternigg et al. 2007), was also included in this study. The HIS-tag purified, recombinant enzymes were analyzed by SDS–PAGE (Figure 1). Unlike the *Drosophila* FDL enzyme, the recombinant *Caenorhabditis* enzymes expressed in *P. pastoris* were active with either *N*- or C-terminal HIS-tags; since the *Caenorhabditis* HEX-2 *N*-terminal HIS-tag was removed upon expression and secretion in *P. pastoris*, the enzyme carrying a C-terminal HIS-tag was used in this study. In comparison with the *Drosophila* enzyme, the quality and the yield of the *Caenorhabditis* enzymes recombinantly expressed in *P. pastoris* was at a high level (Figure 1 and Table I).

In addition to *Drosophila* FDL, we cloned and expressed the *A. mellifera* FDL homolog (the *A. mellifera* FDL DNA sequence described in this paper will appear in DDBJ, ENA and GenBank^®^ Nucleotide Sequence Databases under the accession number KJ786476), an insect FDL enzyme not described to date. After correcting several randomly inserted reverse transcriptase–polymerase chain reaction (PCR) mutations, the modified, purified, recombinant enzyme expressed in both, *P. pastoris* and in insect cells, displayed expected activity toward *p*-nitrophenyl-β-d-*N*-acetylglucosaminide (pNP-β-GlcNAc; Table I, AmFDL).

In order to demonstrate that recombinant enzymes are void of activities toward other monosaccharides, we have used a panel of monoaryl-monosaccharides as substrates for the enzymes. We could detect considerable activity of the recombinant enzymes not only toward to pNP-β-GlcNAc but also to *p*-nitrophenyl-β-d-*N*-acetylgalactosaminide (pNP-β-GalNAc); all *Caenorhabditis* enzymes actually favor pNP-β-GalNAc as a substrate in this experiment (Table II and Supplementary data, Table SI). The insect-derived FDL enzymes were recombinantly expressed in two different hosts (the yeast *P. pastoris* and insect High Five cells), which contrast with respect to their *N*-glycosylation. Nevertheless, the obvious difference in the glycosylation [see *N*-glycosidase F (PNGase F) treated enzymes in Figure 1] appears to have a relatively small effect on the performance of the enzyme toward either pNP-β-GalNAc or pNP-β-GlcNAc substrates (Supplementary data, Table SI).

**Table II.** The activity of purified, recombinant hexosaminidase preparations toward different *p*-nitrophenyl sugars

% activity	DmFDL^a^	DmFDL^b^	AmFDL^a^	AmFDL^b^	CeHEX-2^a^	CeHEX-3^a^	CeHEX-4^a^
α-Glc	*nd*	*nd*	*nd*	<0.20	<0.01	<0.05	*nd*
β-Glc	*<0.05*	<0.50	*<0.05*	<0.30	<0.01	<0.25	<0.02
α-Gal	<0.10	<0.10	<0.20	<0.50	<0.01	<0.10	<0.05
β-Gal	<0.10	<0.10	<0.20	<0.20	<0.05	<1.00	<0.10
α-GlcNAc	*<0.15*	<0.20	<0.50	<1.00	<0.01	<0.50	<0.10
β-GlcNAc	**100.00**	**100.00**	**100.00**	**100.00**	**52.40**	**32.90**	**19.50**
α-GalNAc	*<0.05*	<0.10	<0.50	<1.00	<0.01	<0.10	*<0.10*
β-GalNAc	**17.10**	**17.50**	**27.03**	**23.40**	**100.00**	**100.00**	**100.00**
α-Xyl	*nd*	*nd*	*nd*	<0.10	<0.01	<0.20	*nd*
β-Xyl	*<0.02*	<0.20	<0.10	<0.20	<0.01	<0.20	*nd*
α-Man	*<0.03*	<0.05	<0.10	<0.20	<0.01	<0.25	*nd*

pNP-β-GlcNAc or pNP-β-GalNAc were set to 100% and the remaining values are % relative to the major activity. *nd*, not detected.

^a^Enzymes produced in *P. pastoris*.

^b^Enzymes produced in *Trichoplusia ni* Hi5 cells.

### Properties of pure, recombinant FDL enzymes

**Fig. 2.**
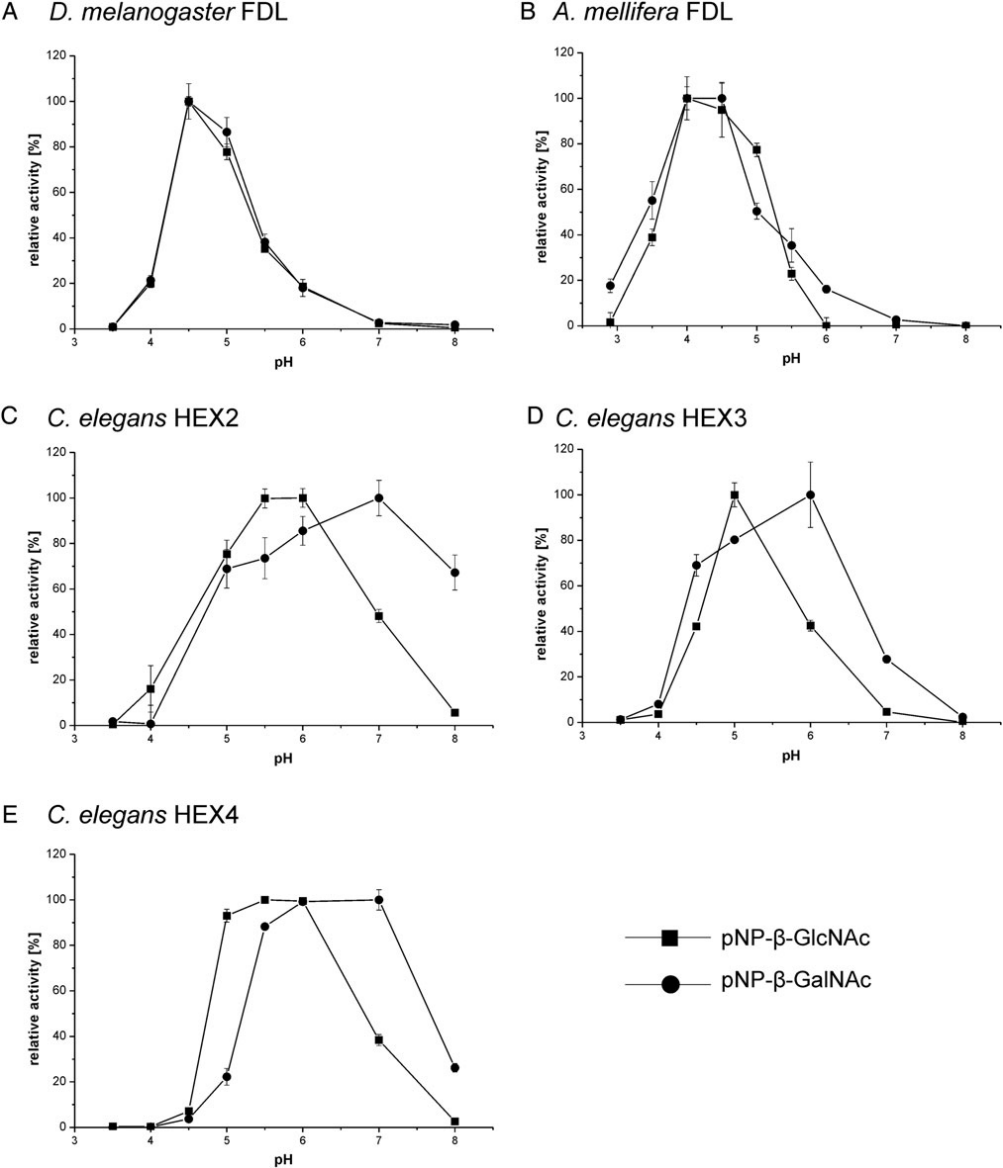
pH optima of the purified, recombinant hexosaminidases. pNP-β-GlcNAc (squares) and pNP-β-GalNAc (circles) were used as substrates. (**A**) *Drosophila melanogaster* FDL, (**B**) *A. mellifera* FDL, (**C**) *Caenorhabditis* HEX-2, (**D**) *Caenorhabditis* HEX-3 and (**E**) *Caenorhabditis* HEX-4. *Drosophila melanogaster* and *A. mellifera* FDL enzymes were expressed in *P. pastoris*. Values represent averages±standard error.

**Fig. 3.**
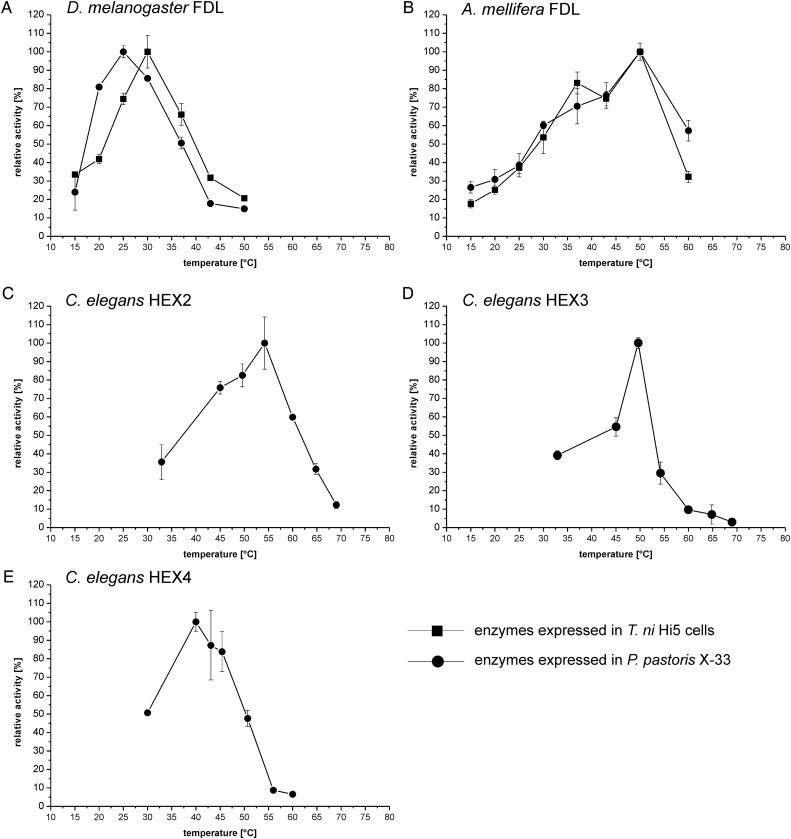
Temperature optima of the purified, recombinant hexosaminidases. pNP-β-GlcNAc was used for FDL proteins from *Drosophila* and *A. mellifera* and pNP-β-GalNAc was used for *Caenorhabditis* hexosaminidases. (**A**) *Drosophila* FDL, (**B**) *A. mellifera* FDL, (**C**) *Caenorhabditis* HEX-2, (**D**) *Caenorhabditis* HEX-3 and (**E**) *Caenorhabditis* HEX-4. For (A) and (B), values for *P. pastoris*-derived enzyme preparations (circles) and insect cell culture-derived enzymes (squares) are shown. Values represent averages±standard error.

The recombinant insect FDL enzymes generally have lower pH optima (pH 4–5) than the *Caenorhabditis* hexosaminidases (pH 5–6) when using either pNP-β-GalNAc or pNP-β-GlcNAc as substrates (Figure 2). Interestingly, as with the *Drosophila* FDL (Léonard et al. 2006), the *Apis* FDL also has higher pH optima toward *N-*glycan substrates than toward pNP-β-GlcNAc/GalNAc; this was not observed for any of the *Caenorhabditis* enzymes described in this study (data not shown). Differences between the recombinant enzymes were also detected when measuring the temperature optima using either pNP-β-GalNAc (*Caenorhabditis* HEX-4) or pNP-β-GlcNAc [all recombinant FDL(-like) enzymes] as substrates; the *Drosophila* FDL enzyme displays the lowest temperature optimum (25–30°C; Figure 3). In addition to testing the recombinant enzymes pH and temperature optima, we have analyzed the sensitivity of the enzymes to presence of free GlcNAc and GalNAc in the reaction mixture. We could show that none of the tested enzymes are inhibited by these monosaccharides, apart from the CeHEX-4 which is inhibited by the GalNAc monosaccharide (*k*_i_ = 1.0 mM; Supplementary data, Figure S3).

The stability of the recombinant, pure FDL enzymes was also assessed. The FDL enzymes remained active with only minor loss of activity (<15%) for at least 6 months at the concentration higher than 100 µg mL^−1^ at 4°C in 20 mM Tris–Cl, 25 mM NaCl, pH 7.5. In contrast to the *Caenorhabditis* enzymes, the insect FDL enzymes appeared to be sensitive to lyophilization (activity loss of 98%) and also to the freeze-thaw procedure (no activity was recovered after one freeze-thaw step).

### The *A. mellifera* hexosaminidase is a true FDL enzyme

**Fig. 4.**
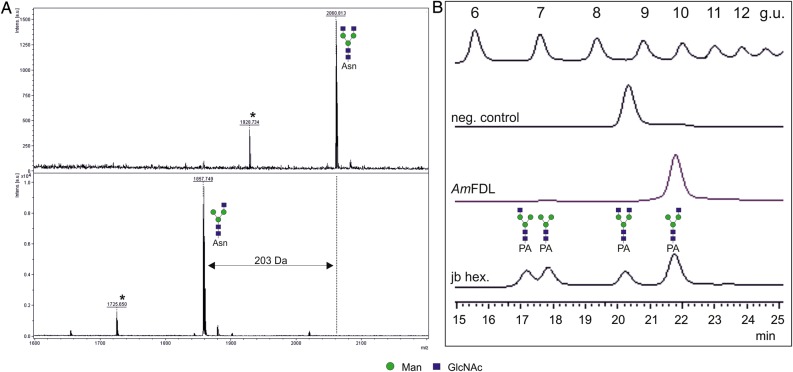
*Apis mellifera* hexosaminidase is a true FDL enzyme. The recombinantly expressed, affinity-purified *A. mellifera* hexosaminidase removes only one of the two terminal β-GlcNAc residues from a glycopeptide carrying typical biantennary *N-*glycan (structures were detected in [M+H^+^] form) (**A**). To confirm whether the recombinant enzyme shows any preference to one or the other terminal β-linked GlcNAc of a biantennary *N-*glycan, the activity of the recombinant enzyme was measured using a 2-aminopyridine modified biantennary *N-*glycan containing two terminal, β-linked GlcNAc residues. For comparison, the same *N-*glycan structure was partially digested in the presence of jack bean hexosaminidase (jb hex.) generating three different products, as described previously (Léonard et al. 2006) (**B**). Asterisk indicates the peaks derived from the laser-induced removal of the dabsyl group from the dabsylated glycopeptides. The glycans are depicted following the glycan nomenclature of the Consortium for Functional Glycomics (http://www.functionalglycomics.org). g.u., glucose units.

The initial analysis of the recombinant *A. mellifera* enzyme demonstrated its activity toward artificial, monoaryl substrates (pNP-β-GlcNAc and pNP-β-GalNAc; Table II). To assess whether the recombinant *Apis* enzyme can process *N-*glycans as substrates, a glycopeptide carrying typical biantennary *N-*glycan terminating with β-linked GlcNAc residues was used (Figure 4A). Moreover, to confirm whether the recombinant enzyme shows any preference to one or the other terminal β-linked GlcNAc of a biantennary *N-*glycan, we have analyzed the activity of the enzyme using a 2-aminopyridine modified *N-*glycan substrate and reversed-phase (RP)-HPLC. We could demonstrate that the enzyme shows the same specificity for the terminal GlcNAc residue of the α1,3-arm of an biantennary *N-*glycan (Figure 4B) as previously reported FDL enzymes.

### Complex substrates of recombinant FDL enzymes

**Fig. 5.**
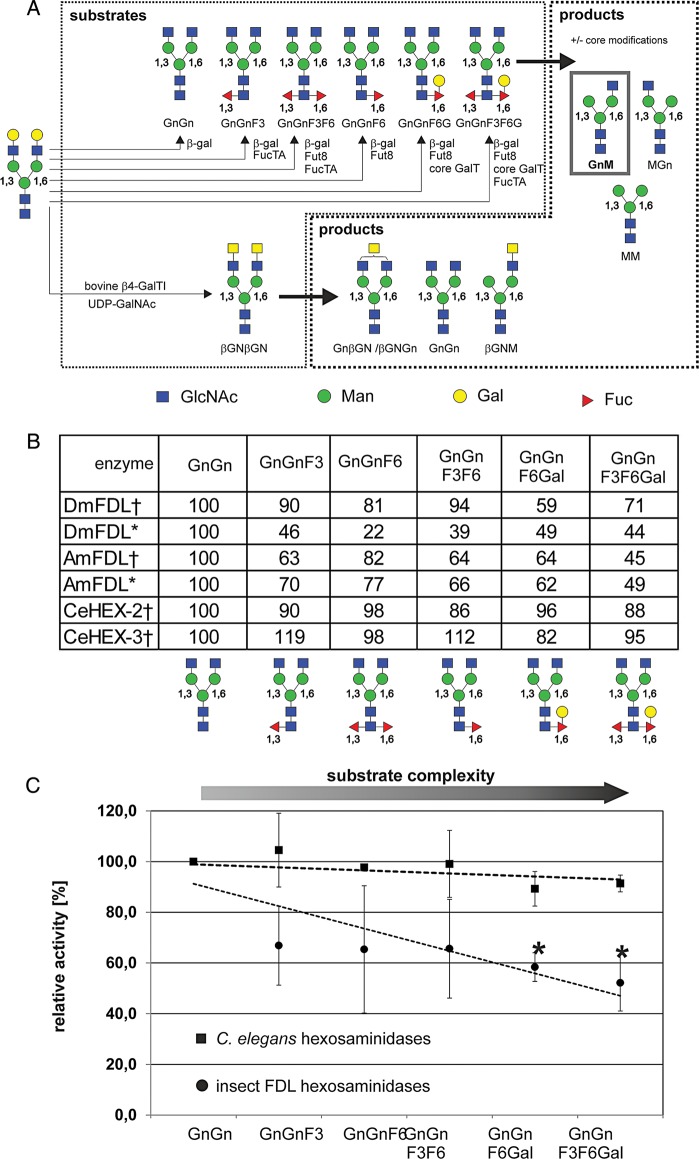
Production of substrates used in this study and the sensitivity of insect FDL enzymes to *N-*glycan core modifications. (**A**) Substrates with and without complex core modifications. Dabsylated-GalGal glycopeptide was incubated with the *Aspergillus nidulans* galactosidase rlacA (Dragosits et al. 2014) in order to remove the galactose residues, followed by treatment with the *A. thaliana* FucTA (Wilson et al. 2001), *C. elegans* FUT-8 (Paschinger et al. 2005) and the recombinant *N-*glycan core β1,4-galactosyltransferase (Titz et al. 2009) as described in the Materials and methods section. An aliquot of the dabsylated-GnGn glycopeptide was treated with bovine β1,4-galactosyltransferase I in the presence of UDP-GalNAc to create the dabsylated-βGNβGN glycopeptide. Enzymes were used sequentially as indicated in order to obtain several complex core modifications. (**B**) Efficiency of *Caenorhabditis* and insect FDL(-like) enzymes for dabsylated-GnGn glycopeptide substrates with core modifications was tested. Values show relative efficiency with dabsylated-GnGn glycopeptide set to 100% efficiency in product formation (dabsylated-GnM) as estimated by the area of the relevant peaks in MALDI-TOF/TOF MS spectra. Average values of duplicate measurements are shown. (**C**) Overall impact of core modifications (substrate complexity) on the activity of *C. elegans* hexosaminidases (HEX-2/3, squares) when compared with insect FDL enzymes (DmFDL and AmFDL, circles). Values represent averages±standard deviation. Linear regressions (dashed lines) were used to depict the trend of substrate conversion. Activities on substrates with a significant decrease (*p* < 0.005) as compared with GnGn are marked with an asterisk. Dagger indicates the enzyme produced in *P. pastoris* and asterisk indicates the enzyme produced in High Five insect cells.

Although estimating the activity of the recombinant glycosidases using monoaryl-substrates is of considerable value, the activity of the enzymes should be, if possible, measured using substrates (nearly) identical to the natural ones. Previous studies described the complex biantennary *N-*glycan structure containing two terminal β-GlcNAc residues and without any other residues linked to the *N-*glycan core as a substrate for the FDL enzymes (Léonard et al. 2006; Geisler et al. 2008; Geisler and Jarvis 2012; Huo et al. 2013). In this study, we have estimated the activity of the recombinant FDL enzymes using various *N-*glycan substrates, which we produced in small amounts using recombinantly produced core-modifying enzymes from previous studies: the substrates used were prepared from dabsylated-GnGn glycopeptide derived from fibrin (Altmann, Schweiszer, et al. 1995) sequentially incubated with the relevant glycosyltransferases [core α1,3-fucosyltransferase (Wilson et al. 2001), core α1,6-fucosyltransferase (Paschinger et al. 2005) and core β1,4-galactosyltransferase (Titz et al. 2009); for the structures and the synthesis scheme, see Figure 5A]. The generated substrates were used to test activities under standard reaction conditions. We could show that all recombinant FDL(-like) enzymes appear to be active toward all tested substrates carrying various combinations of residues at the core of the *N-*glycan (Figure 5B); the *Caenorhabditis* enzymes were less sensitive when compared with the insect FDL enzymes to growing complexity of the *N-*glycan core (e.g. galactosylation of the core-linked fucose, Figure 5C).

Since the recombinant enzymes were able to process pNP-β-GalNAc, we measured the activity of the recombinant enzymes with the dabsylated glycopeptide carrying two terminal β-GalNAc residues (dabsylated-βGNβGN glycopeptide; for the structure, see Figure 5A). Unexpectedly, we could not only detect the activity of the *Caenorhabditis* HEX-2 and HEX-4 (Gutternigg et al. 2007), but also the activity of different FDL(-like) enzymes toward this substrate (Table III). Of the enzymes expressed in *P. pastoris*, in addition to CeHEX-2 and CeHEX-4 (Gutternigg et al. 2007), the purified CeHEX-3 also utilized the dabsylated-βGNβGN glycopeptide as a substrate. Strikingly, *Drosophila* FDL enzymes expressed in insect cells showed very high activity toward this substrate, whereas *Pichia*-derived recombinant proteins did not. Although no contamination was obvious based on SDS–PAGE visual inspection, this result indicated that endogenous hexosaminidases from insect cell cultures were potentially present in the enzyme preparations. Indeed, we observed significant hexosaminidase activity in the culture supernatants of various insect cell lines (Supplementary data, Figure S5). Moreover, we performed a mock-purification of a HIS-tagged non-hexosaminidase protein (Krammer et al. 2012) and observed hexosaminidase activity in activity assays using monoaryl-substrate (Supplementary data, Figure S5). Tests on the βGNβGN glycopeptide substrate confirmed the ability of purified hemagglutinin-preparations derived from *Trichoplusia* ni Hi5 cell cultures to remove two HexNAc residues from the βGNβGN glycopeptide (data not shown). In this context, although higher yields could be achieved for recombinant FDL enzymes in insect cell culture using the baculovirus system, it is important to note that substantial contamination with endogenous hexosamindases might occur and can render such enzymes inapt for enzymatic characterization and structural *N-*glycan analysis.

**Table III.** Enzymatic activity toward dabsylated glycopeptide carrying an biantennary *N-*glycan with two terminal β-GalNAc residues

Enzyme	% βGNβGN	% βGNGn or % GnβGN	% GnGn or % βGNM or % MβGN	% GnM or MGn	% MM
No enzyme control	100.0	0.0	0.0	0.0	0.0
DmFDL^a^	100.0	0.0	0.0	0.0	0.0
DmFDL^b^	29.9	54.3	15.8	0.0	0.0
AmFDL^a^	97.4	0.0	2.6	0.0	0.0
AmFDL^b^	2.1	3.8	18.5	75.6	0.0
CeHEX-2	0.0	0.0	0.0	23.1	76.9
CeHEX-3	0.0	0.0	0.0	100.0	0.0
CeHEX-4	0.0	0.0	100.0	0.0	0.0
JB hexosaminidase	0.7	0.0	10.4	0.5	88.5

Incubations were performed for 16 h. The product formation was quantified by estimating the area of the detected peaks in MALDI-TOF/TOF MS spectra. The corresponding structures of the tested substrates are shown in Figure 5A. JB, jack bean.

^a^Enzymes produced in *P. pastoris*.

^b^Enzymes produced in *Trichoplusia ni* Hi5 cells.

**Fig. 6.**
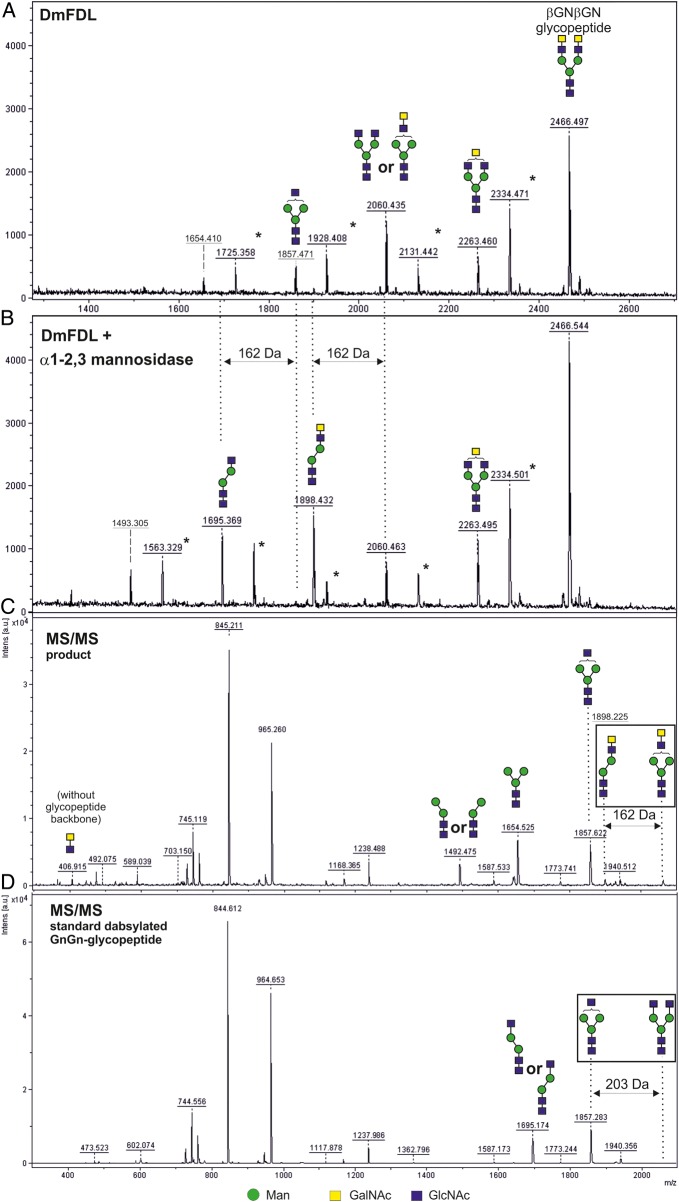
The recombinant DmFDL can process *N-*glycan substrates carrying terminal LacdiNAc. The dabsylated-βGNβGN glycopeptide (containing two terminal β-linked GalNAc residues) was partially processed in the presence of excess of the DmFDL enzyme produced in *P. pastoris* over an extended period of 3 days (**A**). The products were incubated in the presence of α1,3-mannosidase, which facilitated the removal of a terminal, α1,3-linked mannose residue indicating that a part of the DmFDL products are structures lacking one GlcNAc and one GalNAc residues, instead of two GalNAc residues (**B**). This result is corroborated by the presence of the ion with *m/z* 1898 in the MS/MS spectra of the relevant product (the structure with *m/z* 2060) (**C**). In contrast, the MS/MS spectrum of the dabsylated GnGn-glycopeptide carrying one GlcNAc residue on each arm of the biantennary *N-*glycan (the structure with the same *m/z* of 2060) contains neither the ion with *m/z* 407 (corresponding to two linked HexNAc residues) nor the ion with *m/z* 1898 (**D**). All structures were detected in the [M+H^+^] form. The glycans are depicted following the glycan nomenclature of the Consortium for Functional Glycomics (http://www.functionalglycomics.org). Asterisk indicates the peaks derived from the laser-induced removal of the dabsyl group from the dabsylated glycopeptides.

In order to analyze whether *N*-acetylgalactosaminidase activity can also be an intrinsic property of these enzymes when used at higher concentration and/or for longer periods of time, we used FDL enzymes expressed in *P. pastoris* in a second experiment using the dabsylated-βGNβGN glycopeptide as a substrate. In contrast to the results shown in Table III, we have incubated an excess amount (10×) of the recombinant insect FDL enzyme expressed in *P. pastoris* over an extended period of time (3 days) with the dabsylated-βGNβGN glycopeptide as a substrate. We could confirm that the FDL enzymes, under these extreme conditions, could remove terminal β-GalNAc residues. Utilizing α1,3-mannosidase, we could also show that the majority of the structure from which two HexNAc residues had been removed (*m/z* 2060) had these residues attached to the same, α1,3-arm of the *N-*glycan; this result could be corroborated by the appearance of the fragments with the *m/z* 407 and 1898 in the MS/MS analysis of the structure (Figure 6 and Supplementary data, Figure S4) and highlights the high preference of FDL enzymes for the α1,3-arm even under these extreme reaction conditions. Nevertheless, this experiment demonstrated that the FDL enzymes expressed in *P. pastoris* are also able to remove the second GlcNAc residue linked to the α1,6-linked arm of a biantennary *N-*glycan (structure with *m/z* 1654), albeit at a much slower rate when an excess of enzyme was used.

### Amino acid residues critical for the activity of *Drosophila* FDL

**Fig. 7.**
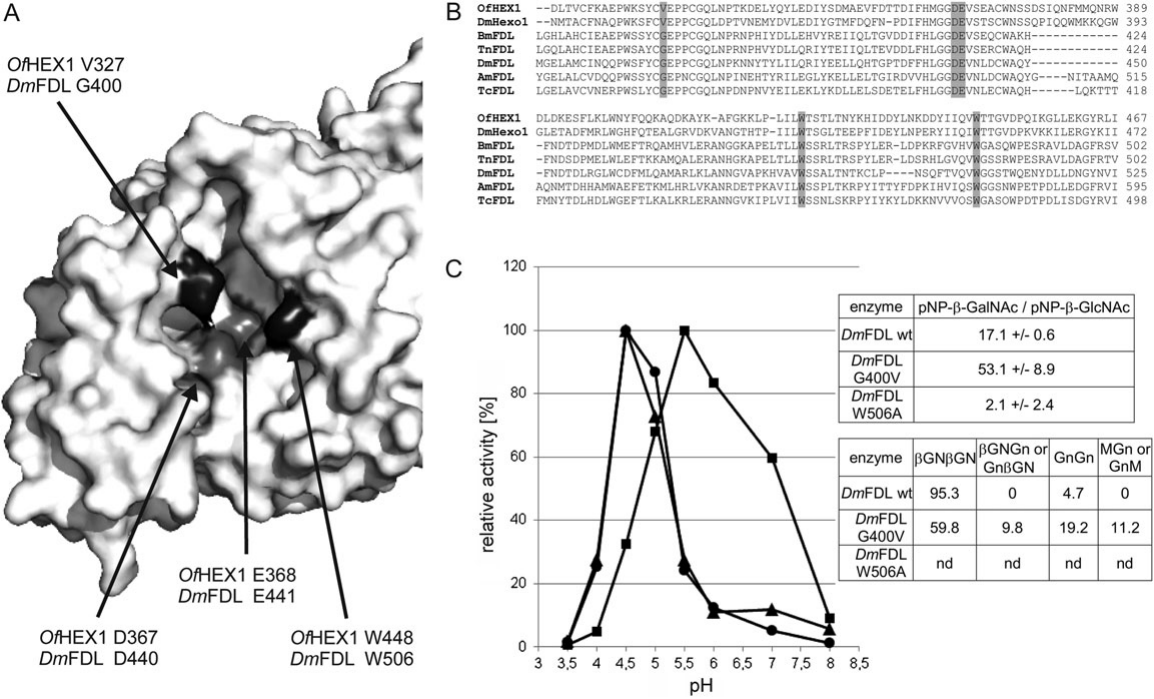
Amino acid residues with importance for the activity of *Drosophila* FDL. (**A**) Active site of the chitinolytic *O. furnacalis* HEX-1 enzyme (PDB ID 3NSM). Amino acid residues with importance for activity (and corresponding residues in the *Drosophila* FDL enzyme) are shown. (**B**) Sequence alignment of two chitinolytic hexosaminidases (OfHex1 and DmHexo1) and verified as well as predicted FDL enzymes. *Of*, *O. furnacalis*; *Dm*, *D. melanogaster*; *Am*, *A. mellifera*; *Bm*, *Bombyx mori*; *Tn*, *Trichoplusia ni*; *Tc*, *Tribolium castaneum*. (**C**) pH optimum of wt and mutant *Dm*FDL (triangles, wt enzyme; circles, W506A; squares, G400V) as well as activity toward pNP-β-GalNAc and βGNβGN glycopeptide. βGNβGN assays were performed for 2 days as described in Material and methods with a undiluted purified enzyme (∼200 ng of protein per assay). Values represent the averages of duplicate measurements. *Nd*, not determined.

In a recent study, the structural basis for the enzymatic activity of a chitinolytic hexosaminidase HEX-1 from the pest *Ostrinia furnacalis (Of*Hex1) was analyzed. In the active site, two well conserved catalytic residues (aspartate 367 and glutamate 368) are surrounded by several conserved tryptophan residues but also a valine residue (V327) that are involved in sugar binding (Figure 7A; Liu et al. 2011). Generally, the amino acid residues in the active site are well conserved among chitinolytic and glycan-processing hexosaminidases (Figure 7B). In the initial study by Liu and colleagues, the authors postulated a large conformational change of the enzyme and reported that the mutation of the “lid” residue tryptophan 448 to alanine led to a drastically decreased catalytic activity of the enzyme (Liu et al. 2011). In a follow-up study, it was reported that in contrast to the wild-type enzyme, the mutation of valine 327 to glycine enabled processing of β1,2-linked GlcNAc (Liu et al. 2012). As can be seen in Figure 7B, D448 is well-conserved among chitinolytic and glycan-processing hexosaminidases, whereas the V327 position of OfHEx1 contains a conserved glycine residue in FDL enzymes from different species.

In order to analyze the effect of these two mutations on FDL enzyme activity, the corresponding residues (W506 and G400) of the codon-optimized DmFDL enzyme were mutated and expressed in *P. pastoris*. Strikingly, the change in the tryptophan residue at position 506 to alanine (W506A) led to massive (more than 200-fold) decrease in enzymatic activity when tested with pNP-β-GlcNAc. In contrast, the mutation of glycine at position 400 to valine (G400V) did not result in the reduction of enzymatic activity. Using pNP-β-GlcNAc, we show that, despite a massive loss of activity, the W506A mutant has a pH optimum similar to the wild-type enzyme, whereas the G400V mutant of DmFDL shows a shift toward a more basic pH (Figure 7C). As mentioned above, all hexosaminidases analyzed in the current study, were able to process pNP-β-GlcNAc and pNP-β-GalNAc, with FDL enzymes from *Drosophila* and *Apis* having a strong preference for pNP-β-GlcNAc. As can be seen in Figure 7C, the *Drosophila* G400V mutant showed increased, and the W506A mutant showed decreased activity toward pNP-β-GalNAc when compared with the wild-type enzyme. We also performed tests on dabsylated-βGNβGN glycopeptide with the wild-type enzyme and the G400V mutant. As described earlier, the wild-type enzyme is able to process βGNβGN under extreme reaction conditions. However, our data indicate that the βGNβGN glycopeptide serves as a better substrate for the G400V DmFDL mutant (Figure 7C). Neither the wild-type nor the G400V mutant was able to process 2-aminopyridine (PA)-labeled chitobiose (data not shown).

Clearly, these data indicate a high structural similarity of chitinolytic and glycan-processing enzymes, since the mutation of W506 of DmFDL led to a similar decrease in activity as the mutation of the corresponding OfHex1 residue. Furthermore, the well-conserved glycine residue (G400 for DmFDL) in FDL enzymes seems to be important for defining their enzymatic specificity.

### Revealing structural features of *Caenorhabditis N-*glycans

**Fig. 8.**
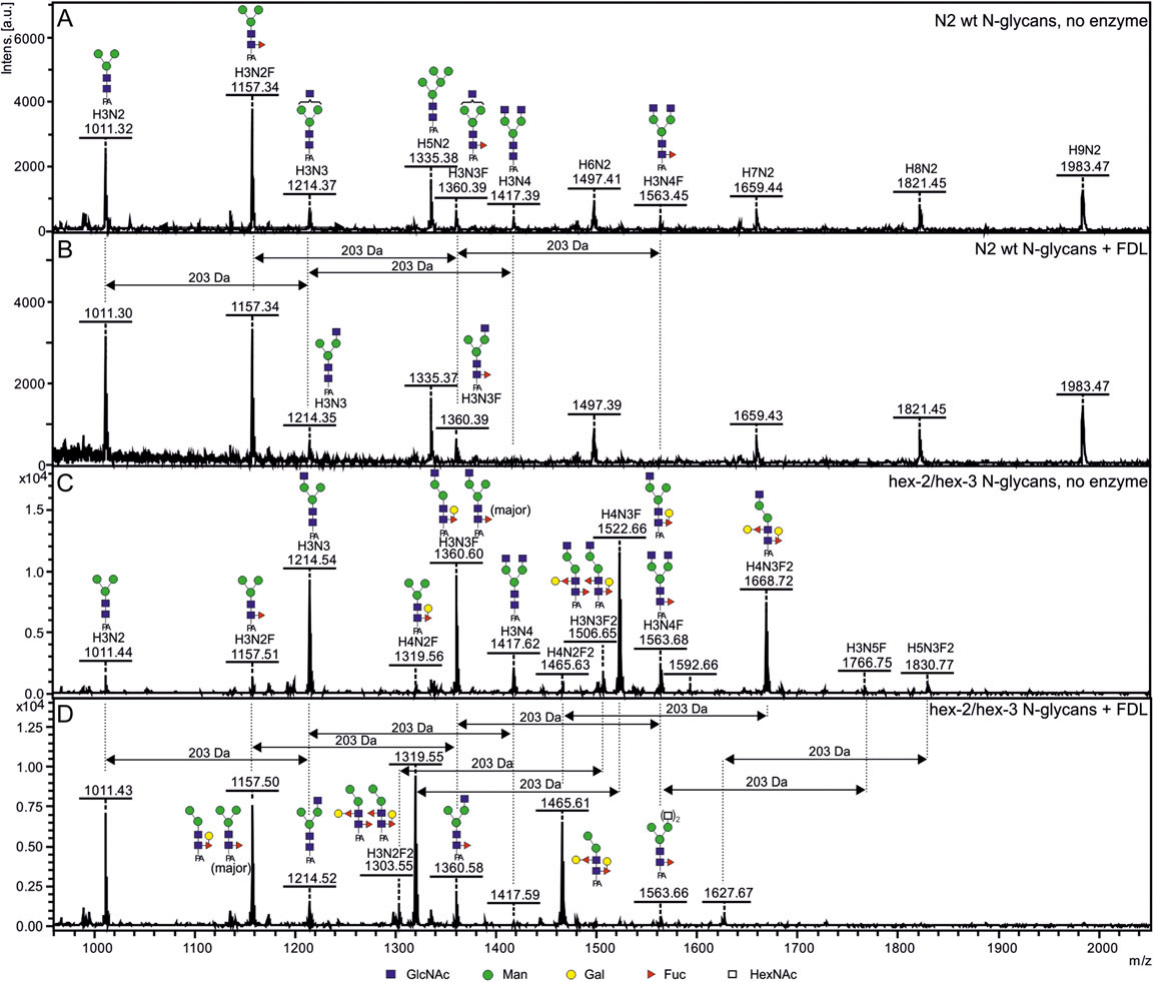
Analysis of *Caenorhabditis* wild-type and mutant *N-*glycans treated with the recombinant DmFDL enzyme. The *N-*glycans of *Caenorhabditis* N2 wild-type and *hex-2*/*hex-3* mutant strains (Yan et al. 2012) were treated with the DmFDL enzyme (produced in *P. pastoris*) for 2 h. The wild-type *N-*glycan structures carrying GlcNAc residues are sensitive to the enzyme, indicating that they carry GlcNAc linked to α1,3-arm of the *N-*glycan core (**A** and **B**). The structures with *m/z* 1214 and 1360 (B) originate from parent structures carrying an additional GlcNAc residue (A). The majority of *N-*glycan structures from the *Caenorhabditis hex-2*/*hex-3* mutant appear sensitive to the DmFDL enzyme (**D**). The processing of the structure with *m/z* 1668 (**C**) indicates that the enzyme is also able to process *N-*glycan structures carrying two GalFuc epitopes (Galβ1,4Fucα1,6 and Galα1,2Fucα1,3). All structures were detected in the [M+Na^+^] form. The glycans are depicted following the glycan nomenclature of the Consortium for Functional Glycomics (http://www.functionalglycomics.org).

The previously performed analysis of *N-*glycans released by *N*-glycosidase A from *Caenorhabditis hex-2*/*hex-3* double mutant has not resolved the exact position of the terminal β-linked GlcNAc residue on many different *N-*glycans containing this residue (Yan et al. 2012), since such analyses would require purification each of the relevant structures and additional HPLC/MS experiments on these purified structures. In addition, various analyses of *N-*glycans from *Caenorhabditis*, a well-studied organism known to carry close to 200 various *N-*glycan structures (Paschinger et al. 2008), out of which several terminate with one or two β-linked non-reducing GlcNAc residues, have not determined the exact position of the terminal, non-reducing GlcNAc residues on these structures (for a review, see Haslam and Dell 2003). In order to address these questions, we have used the DmFDL enzyme (recombinantly expressed in and purified from *P. pastoris*) for analysis of *N-*glycans of the mutant and the wild-type *Caernohabditis N-*glycans. After incubation with the recombinant FDL enzyme, we could show that the *N-*glycan profile retained its intensity and S/N ratio (Figure 8): only the structures containing terminal β-linked GlcNAc residues were affected. Most notably, the amount of the structures terminating with two β-linked GlcNAc residues (structures with *m/z* 1417 and *m/z* 1563 corresponding to Man_3_GlcNAc_4_ and Man_3_GlcNAc_4_Fuc, respectively) were reduced to barely detectable levels after 2 h. Further incubation with the DmFDL enzyme for additional 2–4 h did not change the *N-*glycan spectrum in any way. Oligomannosidic structures (*m/z* 1335 and *m/z* 1983) were used for normalization in order to compare structure amounts in different spectra (data not shown). Based on the amounts of the structures with *m/z* 1214 and *m/z* 1360 in Figure 8A and the structures *m/z* 1417 and *m/z* 1563 in Figure 8B, we can conclude that a significant portion of the Man_3_GlcNAc_3_ and Man_3_GlcNAc_3_Fuc present in wild-type *Caenorhabditis* N2 glycans contain terminal GlcNAc residue on the α1,3-arm of the *N-*glycan. Similarly, the results from the analysis of the FDL-treated *N-*glycans from *Caenorhabditis hex-2*/*hex-3* mutant clearly indicate that the major portion of the bianntenary *N-*glycans carries the terminal β-GlcNAc residues on the α1,3-arm of the glycans (Figure 8C and D). The combined data from the wild-type and mutant *Caenorhabditis* indicate that indeed the low GnTII activity in *Caenorhabditis* is a major reason for the occurrence of monoantennary *N-*glycan structures carrying only single GlcNAc residue on the non-reducing end in this organism.

In addition, this experiment has clearly shown that the DmFDL can process, apart from the expected substrates carrying a core fucose or Galβ1,4Fucα1,6 epitope (Figure 5), an *N-*glycan structure carrying two GalFuc epitopes (Galβ1,4Fucα1,6 and Galα1,2Fucα1,3) linked to the *N-*glycan core (Figure 8C and D, the structure with *m/z* 1668). Interestingly, the structure containing a total of five HexNAc residues (*m/z* 1766; described in Yan et al. 2012) was also processed by the recombinant FDL enzyme in this experiment, indicating that two HexNAc residues are attached to the α1,6-arm and that a single GlcNAc residue is present on the α1,3-arm of this *N-*glycan structure and accessible to the FDL enzyme used.

## Discussion

Most known exo-β-hexosaminidases (EC 3.2.1.52) can process different terminal β-linked GlcNAc residues of different substrates. Although there are hexosaminidases which are able to degrade a very broad range of substrates regardless of origin and type (e.g. jack bean hexosaminidase, Li and Li 1970), the enzymes showing high specificity for a defined, single terminal β-GlcNAc residue were not known until the detection of the FDL hexosaminidases in insect cell extracts (Altmann, Schwihla, et al. 1995). The identification and subsequent analyses of the FDL enzymes has clearly shown that they play a role in *N-*glycan maturation in many invertebrates (Léonard et al. 2006; Rendic et al. 2008; Geisler and Jarvis 2012). Indeed, the FDL enzymes can be compared with O-GlcNAcase, as both β-hexosaminidases are not “simple” catabolic enzymes, but enzymes with a constitutive and regulative role in an organism.

Among the dominant *N-*glycan structures in insects is the MMF^6^ (GlcNAc_2_Man_3_Fuc; Fabini et al. 2001; Rendic et al. 2006; Aoki et al. 2007). The synthesis of this structure requires an ultimate action of a hexosaminidase, since it is known that insect core fucosyltransferases act only on *N-*glycan substrates with a terminal β-GlcNAc residue linked to the α1,3-arm of the *N-*glycan (Fabini et al. 2001; Paschinger et al. 2005; Rendic et al. 2006). On the other hand, one of the first steps in the modification of the complex *N-*glycan structures is the addition of the second GlcNAc residue to an *N-*glycan by GnTII. Similar to insect core fucosyltransferases, all GnTII enzymes described to date utilize only *N-*glycans containing a GlcNAc residue bound to its α1,3-arm (Bendiak and Schachter 1987; Szumilo et al. 1987; Geisler and Jarvis 2012). In addition, previously it was shown that insects can and need to synthesize small amounts of complex *N-*glycans, some of which were shown to contain sialic acid residues (Aoki et al. 2007; Koles et al. 2007). Therefore, a complete and uncontrolled removal of both terminal β-GlcNAc residues from insect *N-*glycans is undesired. The regulation of this important process was not only “left” to competition of a hexosaminidase and the *N*-acetylglucosaminyltransferases—the process control was tightened up by generation of a highly selective enzyme, which is unable to remove the GlcNAc residue added by the GnTII, ensuring that all *N-*glycans processed by the GnTII will have at least one *N-*glycan antenna terminating in either GlcNAc, Gal or sialic acid residue. Hence, one can conclude that the FDL enzyme has a central role, together with GnTI and GnTII in determining the structural fate of the nascent *N-*glycan (Wagner et al. 1996; Fabini et al. 2001; Kim et al. 2009; Geisler and Jarvis 2012). Indeed, as shown in this and previous studies of organisms lacking FDL (Léonard et al. 2006; Yan et al. 2012), the impact of the FDL enzymes on the overall profile of *N-*glycan structures in the studied organisms is high.

A recent study on *Ostrinia furnacalis* FDL has shown that this FDL enzyme utilizes both pNP-β-GalNAc and pNP-β-GlcNAc as substrates (Huo et al. 2013). All enzymes analyzed in this study were also shown to utilize both substrates. Therefore, the name “hexosaminidase”, with which the insect FDL enzymes were initially christened, holds true: the FDL enzymes can (also) process terminal GalNAc residues. Our kinetic data indicate similar affinities in the low mM range for both GlcNAc and GalNAc residues, although insect FDL enzymes, in contrast to nematode enzymes, displayed lower specific activity toward GalNAc substrates (Supplementary data, Table SI). Although we were able to push in vitro reactions with insect FDL enzymes toward the removal of GalNAc moieties from *N-*glycan substrates, this activity appears to bear little if any relevance in vivo. Our data on mutant DmFDL enzymes indicate a high structural similarity of insect chitinolytic and glycan-degrading hexosaminidases. Although the mutation of a conserved glycine residue of *Drosophila* FDL led to a relaxed enzymatic specificity, it does not seem to be the sole amino acid residue that is important for the strict specificity of these enzymes. Taken together and considering that the synthetic aryl substrates are considerably smaller than complex *N-*glycan substrates, the specificity of insect FDLs may be obtained through steric hindrance, which impairs efficient binding of substrates such as a βGNβGN glycan in vivo.

Previously, it was shown that the *N-*glycan processing *Caenorhabditis* hexosaminidases, although displaying the *N-*glycan specificity of the insect FDL enzymes, are only distantly related to the insect hexosaminidases (Gutternigg et al. 2007) and actually are in a separate clade of the GH20 family (Diez et al. 2005). Again, the comparison of the kinetic parameters of the *Apis* and *Drosophila* FDL enzymes and *Caenorhabditis* hexosaminidases clearly shows the difference in preference toward different aryl substrates (Table II and Supplementary data, Table SI) and thus, further supports this observation. Moreover, a difference in processing of large, *N-*glycan substrates is indicated, whereby the enzyme sensitivity to growing complexity of the *N-*glycan core was significantly higher for insect than for the *Caenorhabditis* enzymes, with insect enzymes being sensitive to any modification of the core in vitro, despite the existence of α1,3 and α1,6-fucose core modifications in vivo (Figure 5). Therefore, our data suggests that the insect FDL enzymes and the *Caenorhabditis* FDL-like hexosaminidases, apart from their mutual FDL-like specificity toward *N-*glycans, are otherwise considerably different and either share one, from an evolutionary standpoint very distant, ancestral hexosaminidase gene or are even products of convergent evolution. In this context, our biochemical data may support a previous phylogenetic analysis that indicated a functional shift upon gene duplication in the evolution of FDL enzymes in insect cells and more generally also concluded that the evolution of the synthesis of paucimannosidic *N-*glycans may have occurred independently on multiple occasions (Intra et al. 2008).

Since the previous studies (Léonard et al. 2006; Gutternigg et al. 2007) have not analyzed all parameters relevant for the recombinant FDL(-like) enzymes and have directly used recombinant proteins present in *P. pastoris* culture supernatants, we have also analyzed the temperature optima of the purified, recombinant enzymes. Unexpectedly, all *Caenorhabditis* enzymes and *Apis* FDL have, when compared with *Drosophila* FDL, relatively high temperature optima. However, it appears that the different *N*-glycosylation of the insect-derived FDL enzymes, produced in different expression systems (see the difference in the estimated size of proteins; Figure 1), has only a minor effect on their pH/temperature optima and kinetic properties when using small, monoaryl substrates (Supplementary data, Table SI, Figures 3 and S6). It should be noted that, as shown for other FDL enzymes (Léonard et al. 2006; Huo et al. 2013), the pH optima of the insect FDL enzymes described in this study was higher toward larger, natural substrates than toward smaller, artificial substrates. The pH optima for *N-*glycan substrates are in the range expected for enzymes in the Golgi apparatus with mildly acidic conditions of about pH 6.0–6.5.

Analyses of *N-*glycan structures terminating with β-GlcNAc residues often do not delve into the exact determination of the position of the terminal GlcNAc residues on biantennary *N-*glycans, since such structural evaluations require additional HPLC runs of (purified) structures or MS^n^ analyses of chemically modified *N-*glycan structures. In this study, we clearly demonstrate the successful use of the highly specific and pure DmFDL enzyme in the determination of the position of the terminal β-GlcNAc residues in complex mixtures of *N-*glycans. In this context, our data also shed light on the report of *N-*glycan structures of *Caenorhabditis* wild-type and *hex-2*/*hex-3* mutant strains, clarifying the relative amounts of structures containing the terminal β-GlcNAc bound to the α1,6-arm of *N-*glycans. Indeed, a number of substrates decorated with various additional sugar moieties are utilized by these highly selective hexosaminidases. In addition, although bisecting bi- or tri- or tetraantennary *N-*glycans appear not to be susceptible to *Bombyx mori* FDL hexosaminidase (Nomura et al. 2010), our preliminary data on a triantennary structure carrying three terminal β-linked GlcNAc residues show that *Drosophila* FDL can act on these substrates (data not shown). Such interpretations are not possible with other typical enzymes used for structural glycan analysis such as jack bean hexosaminidase due to their “general” specificity. It is, therefore, intriguing to exploit the specificity of recombinantly produced FDL enzymes in order to support structural *N-*glycan analysis. The current understanding of the substrate spectrum of the FDL(-like) enzymes is summarized in Table IV.

**Table IV.** Substrate preference of invertebrate FDL(-like) hexosaminidases characterized to date in comparison with selected *N-*glycan modifying hexosaminidases

Substrate type/complexity	Monosaccharide (aryl)*	Monosaccharide (aryl)*	Biantennary *N-*glycan	Biantennary *N-*glycan with or without core fucose	Biantennary *N-*glycan with two or three core residues
Substrate	*p*-nitrophenyl-β-GlcNAc*	*p*-nitrophenyl-β-GalNAc*	βGNβGN-glycan/GP	GnGn-glycan/GP [±F^3^ or F^6^]	GnGnF^6^Gal- or GnGnF^6^F^3^Gal- glycan/GP
*C. elegans* HEX-2	+^1^	+^1^	+^1^	+^1^	+
*C. elegans* HEX-3	+^1^	+^1^	+^1,6^	+^1^	+
*C. elegans* HEX-4	+^1^	+^1^	+^1^	−	−
*D. melanogaster* FDL	+^2^	+	−	+^1^	+
*A. mellifera* FDL	+	+	−	+	+
*C. elegans* HEX-5^1^	+	+	+	−	n/d
*B. mori* FDL^3^	+	n/d	n/d	+	n/d
*O. furnacalis* FDL^5^	+	+	n/d	+	n/d
*S. frugiperda* FDL^4^	+	n/d	n/d	+	n/d

The analyses performed in this study are labeled with grey background. The structures, which are known or expected to be biologically relevant substrates for the tested enzymes, are labeled with darker grey background. *, non-natural substrate; n/d, not determined; “+”, activity detected; “−”, activity not detected; GP, glycopeptide.

^1^(Gutternigg et al. 2007); ^2^(Léonard et al. 2006); ^3^(Nomura et al. 2010); ^4^(Geisler et al. 2012); ^5^(Huo et al. 2013); ^6^Activity of CeHEX-3 toward dabsylated βGNβGN-glycopeptide was previously not detected when using *P. pastoris* culture supernatants (Gutternigg et al. 2007).

Although all FDL(-like) hexosaminidases share the specificity for the terminal β-linked GlcNAc residue linked to the α1,3-arm of a biantennary *N-*glycan, several differences in substrate utilization emphasize the phylogenetic distance between the insect and nematode enzymes. Given this relatively large phylogenetic distance of insect and nematode hexosaminidases (see also Gutternigg et al. 2007), it will be interesting to investigate key amino acid residues of invertebrate glycan-processing hexosaminidases in more comprehensive studies on engineered forms of these enzymes, which will aid understanding of their biochemical and biological function.

## Materials and methods

### Molecular cloning procedures

For cloning, ORFs were obtained by PCR (Dynazyme EXT polymerase, Thermo Scientific, USA) with primers listed in Supplementary data, Table SII. For *D. melanogaster* and *A. mellifera* FDL, cDNA templates were used, whereas for *C. elegans* HEX genes, expression vectors as originally described by Gutternigg et al. (2007) were used as PCR templates. For expression in *P. pastoris*, either a standard pPICzαA vector for C-terminal myc/HIS-tag fusion or a modified pPICzαA encoding for *N*-terminal HIS-/Flag-tag sequence was used (Nemcovicova et al. 2013). For the baculovirus insect cell expression system, a modified pFastBac Dual vector backbone with the melittin secretion signal sequence (Tessier et al. 1991) and an *N*-terminal HIS-tag encoded downstream of the polyhedrin promoter was used as expression backbone. For codon-optimized expression of *Drosophila* FDL, a *P. pastoris* codon-optimized sequence was custom synthesized by GenScript, USA. PCR products/ORFs and vector backbones were digested with the respective restriction enzymes (New England Biolabs, USA), ligated (T4 Ligase, New England Biolabs, USA) and positive clones were selected on LB plates containing either 25 µg mL^−1^ zeocin for *P. pastoris* constructs or 50 µg mL^−1^ ampicillin for baculovirus constructs. For the generation of *Drosophila* FDL mutants, mismatch primers (Supplementary data, Table SII) in combination with Pfu hotstart Cx polymerase (Agilent, USA) were used. After PCR amplification, the plasmid template was digested using DpnI and the purified PCR product was used for transformation of *Escherichia coli. Escherichia coli* JM109 cells were used for all cloning steps. DNA sequences were verified by Sanger sequencing. All constructs were designed without the original transmembrane domains; the *Saccharomyces c*e*revisiae* α-mating factor or melittin signal sequences were used for secreted expression in *P. pastoris* or insect cells, respectively.

### Recombinant protein expression

pPIC vector constructs for *P. pastoris* expression were linearized and used to transform *P. pastoris* X-33 competent cells followed by selection on YPD plates containing 100 µg mL^−1^ zeocin. After selection of positive clones, 6–10 individual clones were cultivated in 2 mL YP medium at 28°C, 200 rpm overnight. Cells were collected, resuspended in 10 mL YP medium containing 1% methanol and grown at 25°C, 200 rpm for 48 h. The cultures were supplemented with an additional 1% methanol after 24 h. Expression of recombinant hexosaminidases was verified by Western blotting and pNP-β-GlcNAc- and/or -GalNAc activity assays as described below. For each construct, the clone with the highest activity was used for expression in a larger volume (200 mL YP, 1% methanol, 25°C, 200 rpm, for 72 h, including adding methanol to 1% after 24 and 48 h, respectively) and culture supernatants were used for subsequent protein purification.

High cell density *P. pastoris* bioreactor cultivation was performed in a Minifors (Infors HT, Switzerland) bioreactor: 200 mL *P. pastoris* culture were used to inoculate 1.5 L glycerol minimal medium (Gasser et al. 2010). After a batch phase of ∼24 h (25 g L^−1^ yeast dry mass), a linear glycerol fed-batch was initiated for 6 h, followed by a methanol pulse and a methanol adaption phase. A linear methanol fed-batch phase was performed for 60 h (final biomass yield 115 g L^−1^; Hohenblum et al. 2004). Temperature was set to 25°C, pH maintained at 5.0 (by the addition of ammonia) and diluted oxygen concentration was maintained at 20% throughout the fermentation. Hexosaminidase activity in the culture supernatant was determined using a synthetic substrate as described below.

For expression in the baculovirus insect cell system, pFastBac Dual constructs (positive clones selected on LB plates containing 100 µg mL^−1^ ampicillin) were used to transform *E. coli* DH10multibac cells and positive bacmid integrations were selected on LB plates containing kanamycin (50 µg mL^−1^), tetracycline (10 µg mL^−1^), gentamycin (7 µg mL^−1^), 5-bromo-4-chloro-3-indolyl-β-d-galactopyranoside (100 µg mL^−1^) and isopropyl β-d-1-thiogalactopyranoside (40 µg mL^−1^) (Bieniossek et al. 2012). Bacmid DNA was purified form *E. coli* cells (DNA Midi-preps, Qiagen, Netherlands) and used for transfection of *S. frugiperda* Sf9 cells using Cellfectin II reagent (Invitrogen, USA) according to the manufacturer's guidelines. Baculovirus was propagated in Sf9 cells and baculovirus stocks were used to infect *Trichoplusia ni* High Five cells. For expression, High Five cells were infected at a multiplicity of infection of ∼5 and cultivated in IPL-41 custom medium at a confluency of ∼30% (T175 flasks, 25 mL culture volume) at 27°C for 2–4 days (Palmberger et al. 2012). Expression of recombinant proteins was verified by Western blotting [anti-polyHistidine IgG from mouse, Sigma 1:3000 and anti-mouse IgG (Fc specific) AP conjugate from goat, Sigma 1:10,000] and pNP-β-GlcNAc assays as described in the section “Enzyme activity assays using pNP-β-GlcNAc and pNP-β-GalNAc”. Culture supernatants were collected and used for protein purification.

### Protein purification

For recombinant protein purification, *P. pastoris* and insect cells were removed by centrifugation at 4°C. The cell culture supernatants were concentrated by an Amicon Ultrafiltration unit (Millipore, Germany) with a 30 kDa NMWL (nominal molecular weight limit) filter (Millipore), followed by buffer exchange to Ni-NTA (nickel-nitrilotriacetic acid) column loading buffer (20 mM Tris, 20 mM NaCl, 5 mM imidazole, pH 8.5). Protein containing solutions were mixed with 1 mL pre-equilibrated Ni-NTA Agarose slurry (Qiagen, Netherlands) and incubated on a rotator for 1 h. The mixture was transferred to a plastic column and after five washing steps with loading buffer containing 20 mM imidazole (2 column volumes per step) and recombinant proteins were eluted with 500 mM imidazole (1 column volume per elution fraction). Purity of the recombinant enzymes was evaluated by SDS–PAGE followed by Coomassie Brilliant Blue staining of the gels. Pure and active fractions were pooled and buffer was exchanged using Vivaspin columns (30 kDa NMWL, Millipore, Germany) in order to transfer the protein into a storage buffer (20 mM Tris–Cl, 25 mM NaCl, pH 7.5) and to remove imidazole. All recombinant proteins were stored at 4°C and, as such, remained active with only minor loss of activity (<15%) for at least 6 months.

### Determination of protein concentration

Protein concentration of purified products was measured after the removal of imidazole using a BCA (bicinchoninic acid) protein quantification kit (Sigma, USA) with bovine serum albumin as a standard protein.

### Removal of *N-*glycans using PNGase F

Approximately 2.5 µg of purified recombinant protein was supplemented with 1% SDS and incubated at 95°C for 5 min. After cooling on ice, 1.0 µL PNGase F (NEB, USA) or water was added as well as buffer (final concentration of 50 mM sodium phosphate, pH 7.5) and 1% Nonidet P-40 detergent in a total volume of 20 µL. Reactions were incubated at 37°C for 18 h before Tris-Glycine SDS–PAGE (resolving gel: T12.5/C1, stacking gel: T5.7/C2.2) and Coomassie Brilliant Blue G-250 staining were performed.

### Enzyme activity assays using pNP-β-GlcNAc and pNP-β-GalNAc

Standard enzymatic activity assays were performed using pNP-β-GlcNAc (Sigma, USA) and pNP-β-GalNAc (Sigma, USA) as a substrate. The assays were performed at a substrate concentration of 5 mM in McIlvaine buffer, pH 4.0–6.0 at 30°C for *Drosophila* FDL and at 37°C for the other hexosaminidases. The total reaction volume was 40 µL and incubation time was 2 h. All purified enzymes were tested for additional activities using a range of *p*-nitrophenyl-sugars as substrates at a concentration of 5 mM for 4 h. After the addition of 200 µL of 0.4 M glycine/NaOH pH 10.4, A_405nm_ was determined on a Tecan Infinite M200 microtiter plate reader (Tecan, Austria). All enzymes were appropriately diluted for measurements in order to measure reactions within the linear range of the assay. Enzyme units are given in µmol product (nitrophenol) per minute. Specific activity is defined as units per milligram purified protein.

### Determination of the pH optimum, temperature optimum and temperature stability

Tests were performed as described in the previous section at 30 and 37°C with 5 mM pNP-β-GlcNAc and pNP-β-GalNAc as a substrate. McIlvaine buffers with a pH range from 3.0 to 8.0 were used. Tests for the determination of temperature optimum were performed with 5 mM pNP-β-GlcNAc/pNP-β-GalNAc at different temperatures in a Gradient PCR Thermocycler (Eppendorf, Germany) or thermomixers in McIvaine buffer with the pH as determined to be optimal for the specific hexosaminidase**.**

### Determination of *K*_m_, specific activity, *k*_cat_ and *K*_i_

Standard assays were performed in McIlvaine buffers with optimal pH for the respective enzyme at 30 and 37°C. Substrate concentrations in the range of 0.1–10 mM pNP-β-GlcNAc or pNP-β-GalNAc were used. Michaelis constant (*K*_m_), specific activity and *k*_cat_ were calculated using the non-linear curve fitting function available in the OriginPro 8 software package. For the inhibition of the enzyme with GlcNAc and GalNAc, assays were supplemented with GlcNAc or GalNAc at concentrations ranging from 1 to 100 mM. IC50 was calculated based on exponential curve fitting using OriginPro. *K*_i_ was calculated based on the Cheng-Prusoff equation: $K_{i}=\text{IC}50/(1+[S]/K_{m})$ with IC50 being the half maximal inhibitory concentration of GalNAc, [*S*] the pNP-β-GalNAc concentration (5 mM) and *K*_m_ the Michaelis–Menten constant. For Dixon plots, standard assays were performed at 37°C with pNP-β-GalNAc concentrations of 0.73, 1.25, 2.5 and 5 mM and supplemented with GalNAc in the range of 0–10 mM.

### Glycan remodeling of glycopeptide substrates

Dabsylated-GalGal [Gal-β1,4-GlcNAc-β1,2-Man-α1,6(Gal-β1,4-GlcNAc-β1,2-Man-α1,3)Man-β1,4-GlcNAc-β1,4-GlcNAc-β1-Asn] glycopeptide derived from bovine fibrin, possessing the peptide sequence GENR (Gly-Glu-Asn-Arg) (Altmann, Schweiszer, et al. 1995) was used as the starting material in order to obtain complex hexosaminidase substrates (for structures and synthesis scheme, see Figure 5A). Glycan remodeling was essentially performed as described previously (Mucha et al. 2004). In short, galactose moieties were removed by recombinant *A. nidulans* galactosidase A (Dragosits et al. 2014) in 2-(*N*-morpholino)ethanesulfonic acid (MES) buffer pH 6 (80 mM) at 37°C. The fucose α1,3-linked to the proximal GlcNAc of the *N-*glycan core was added using recombinant *A. thaliana* FucTA (Wilson et al. 2001), whereas the α1,6-linked core-fucose was added by using recombinant *Caenorhabditis* FUT-8 (Paschinger et al. 2005). The fucosyltransferase reactions were performed in MES buffer pH 6.5 (80 mM) with a final concentration of 20 mM MnCl_2_ and 1.6 mM guanosine 5′-diphospho-β-l-fucose. The recombinant *N-*glycan core β1,4-galactosyltransferase (Titz et al. 2009) was used to add the galactose residue on the core α1,6-linked fucose in 80 mM MES pH 6.5 supplemented with MnCl_2_ and UDP-galactose at final concentrations of 20 mM and 1.6 mM, respectively. The dabsylated-βGNβGN (containing two terminal β-linked GalNAc residues) was prepared as described previously (Mucha et al. 2004). Briefly, the dabsylated-GnGn glycopeptide was incubated for 65 h at 37°C with 0.6 units of bovine β1,4-galactosyltransferase (Fluka, Buchs, Switzerland) in the presence of 9 mM UDP-GalNAc, 80 mM Tris (pH 7.5) and 10 mM MnCl_2_ (exploiting the ability of bovine milk β1,4-galactosyltransferase to also utilize UDP-GalNAc as a donor). All other glycosyltransferase reactions were performed at room temperature until complete conversion of the substrate (after 16–24 h) was achieved as estimated by an Autoflex Speed matrix-assisted laser desorption/ionization (MALDI)- time of flight (TOF)/TOF MS (Bruker, USA) in positive ion mode using 1% α-cyano-4-hydroxycinnamic acid as a matrix. The glycopeptide substrates were then purified and quantified by RP-HPLC (Shimadzu, Japan) using a C18 column (ODS Hypersil), using 50 mM ammonium acetate pH 6 with a 19–22% acetonitrile/3.2–3.7% isopropanol gradient at a flow rate of 1.5 mL/min. Dabsylated glycopeptides were detected by measuring the absorbance at 500 nm.

### Enzyme assays using dabsylated glycopeptides and PA-labeled glycans

Reactions were performed with 1 nmol glycopeptide, 2 µL McIlvaine buffer, 0.3 µL enzyme and water added to a final volume of 3.5 µL. Enzymatic reactions for DmFDL were performed at pH 5.5 and 30°C, for AmFDL at pH 5.0 and 37°C. For CeHEX-2, 3 and 4 reactions were performed at pH 6.0 and 37°C. Dabsylated-βGNβGN (glycopeptide carrying two terminal β-linked GalNAc residues) reactions were carried out with excess enzyme (∼2.5 µUnits) for 16 h. In the case of DmFDL and AmFDL, the reactions were performed for a total of 3 days with the addition of excess enzyme every 24 h. Subsequently, 0.5 µL of a dabsylated-βGNβGN glycopeptide reaction mixtures were incubated with 0.25 µL α1,2/3 mannosidase (NEB) and 2 µL McIlvaine buffer pH 5.5 in a total volume of 2.5 µL at room temperature over night. Reactions with dabsylated-GnGn (±core modifications) glycopeptide were performed with enzyme dilutions in order to achieve ∼50% substrate conversion within 2 h. Reactions with 2-aminopyridine labeled glycans (PA-glycans) from *C. elegans* (prepared as described previously using PNGase A (Paschinger et al. 2012); for wild type, mixed stages were used, whereas, for the mutant, the predominantly L4 stage was performed by combining 1 µL PA-glycans, 0.5 µL 100 mM ammonium acetate buffer pH 5.0 and 0.5 µL DmFDL enzyme. The reactions were incubated at 30°C for 2 h. All enzymatic reactions were analyzed by an Autoflex Speed MALDI-TOF/TOF MS in positive ion mode using either 1% α-cyano-4-hydroxycinnamic acid (glycopeptides) or 0.3% 6-aza-2-thiothymine (PA-labeled glycans) supplemented with 60 mM NaCl as a matrix.

## Authors’ contributions

MD performed the cloning, expression, purification and characterization experiments. SY performed several MALDI-TOF/TOF MS experiments and prepared *Caenorhabditis N-*glycans. IBHW and ER-F assisted in the experimental design and data evaluation. MD, IBHW and DR wrote the manuscript. IBHW and DR conceived the study.

## Supplementary data

Supplementary data for this article is available online at http://glycob.oxfordjournals.org/.

## Funding

This work was funded by the Austrian Science Fund (FWF) [TRP127 to DR and P23922 to IBHW]. Funding to pay the Open Access publication charges for this article was provided byAustrian Science Fund (FWF).

## Conflict of interest

None declared.

## Abbreviations

BCA, bicinchoninic acid; FDL, fused lobes; GalNAc, *N*-acetylgalactosamine; GlcNAc, *N*-acetylglucosamine; GnTI, *N*-acetylglucosaminyltransferase I; HPLC, high performance liquid chromatography; *k*_cat_, turnover number; *k*_i_, inhibitor binding affinity; *K*_m_, Michaelis–Menten constant; MALDI, matrix-assisted laser desorption/ionization; MES, 2-(*N*-morpholino)ethanesulfonic acid; MS^n^, mass-spectrometric; Ni-NTA, nickel-nitrilotriacetic acid; NMWL, nominal molecular weight limit; ORF, open reading frame; PA, 2-aminopyridine; PCR, polymerase chain reaction; PNGase A/F, *N*-glycosidase A or F; pNP-β-GalNAc, *p*-nitrophenyl-β-d-*N*-acetylgalactosaminide; pNP-β-GlcNAc, *p*-nitrophenyl-β-d-*N*-acetylglucosaminide; SDS–PAGE, sodium dodecyl sulphate–polyacrylamide gel electrophoresis; TOF, time of flight.

## Supplementary Material

Supplementary Data
