# Supplementary material for: Enzymatic properties and subtle differences in the substrate specificity of phylogenetically distinct invertebrate N-glycan processing hexosaminidases
Source: Glycobiology. 2014 Dec 8;25(4):448–64. doi: 10.1093/glycob/cwu132 (PMC4339880; doi:10.1093/glycob/cwu132)
Supplement: Supplementary Data [file supp_25_4_448__index.html]

Enzymatic properties and subtle differences in the substrate specificity of phylogenetically distinct invertebrate N-glycan processing hexosaminidases — Enzymatic properties and subtle differences in the substrate specificity of phylogenetically distinct invertebrate N-glycan processing hexosaminidases — Enzymatic properties and subtle differences in the substrate specificity of phylogenetically distinct invertebrate N-glycan processing hexosaminidases — Supplementary Data 

# Enzymatic properties and subtle differences in the substrate specificity of phylogenetically distinct invertebrate *N*-glycan processing hexosaminidases

## Supplementary Data

Supplementary Data

**Files in this Data Supplement:**

- Supplementary Data - Docx file
