# Supplementary material for: Enzymatic properties and subtle differences in the substrate specificity of phylogenetically distinct invertebrate N-glycan processing hexosaminidases
Source: Glycobiology. 2014 Dec 8;25(4):448–64. doi: 10.1093/glycob/cwu132 (PMC4339880; doi:10.1093/glycob/cwu132)
Supplement: Supplementary Data [file supp_cwu132_cwu132supp.docx]

**Enzymatic properties and subtle differences in the substrate specificity of phylogenetically distinct invertebrate N-glycan processing hexosaminidases**

**Martin Dragosits^1,2^, Shi Yan^1^, Ebrahim Razzazi-Fazeli^2^, Iain B. H. Wilson^1^, Dubravko Rendic^1^***

**SUPPLEMENTARY DATA**

^1^ University of Natural Resources and Life Sciences, Department of Chemistry, Vienna, Austria

^2^ University of Veterinary Medicine, VetCore Facility for Research, Vienna, Austria

**Keywords**:

*Fused lobes* / insect / N-glycans / hexosaminidase / recombinant

* Corresponding author contact information:

Dubravko Rendic, University of Natural Resources and Life Sciences, Vienna, Department of Chemistry, Muthgasse 18, Vienna 1190, Austria. Tel: (+43) 1 / 47654-2176, Fax: (+43) 1 / 47654-6076, e-mail: [dubravko.rendic@boku.ac.at](mailto:dubravko.rendic@boku.ac.at)

**Supplemental Tables**

a)

| **pNP-β-GlcNAc assays** | *K*_m_  **[mM]** | **specific activity**  **[µmol min^-1^ mg ^-1^]** | ***k*_cat_**  **[sec^-1^]** | ***k*_cat_ /** *K*_m_  **[mM^-1^ sec^-1^]** |
| --- | --- | --- | --- | --- |
| DmFDL^†^ | 1.40 +/- 0.08 | 13.4 +/- 0.5 | 48.2 +/- 2.2 | 42.0 +/- 1.8 |
| DmFDL* | 1.17 +/- 0.40 | 9.8 +/- 1.4 | 11.4 +/- 0.3 | 11.2 +/- 0.1 |
| AmFDL^†^ | 0.19 +/- 0.04 | 2.8 +/- 0,1 | 3.6 +/- 0.1 | 16.2 +/- 2.1 |
| AmFDL* | 0.18 +/- 0.03 | 4.6 +/- 0.2 | 5.5 +/- 0.4 | 32.9 +/- 2.6 |
| CeHEX-2^†^ | 0.23 +/- 0.15 | 6.1 +/- 0.5 | 8.6 +/- 0.8 | 22.0 +/- 3.6 |
| CeHEX-3^†^ | 0.97 +/- 0.70 | 0.8 +/- 0.2 | 0.1 +/- 0 | 0.2 +/- 0.1 |
| CeHEX-4^†^ | 3.47 +/- 0.35 | 3.6 +/- 0.3 | 12.6 +/- 1.4 | 2.2 +/- 0.1 |

b)

| **pNP-β-GalNAc assays** | *K*_m_  **[mM]** | **specific activity**  **[µmol min^-1^ mg ^-1^]** | ***k*_cat_**  **[sec^-1^]** | ***k*_cat_ /** *K*_m_  **[mM^-1^ sec^-1^]** |
| --- | --- | --- | --- | --- |
| DmFDL^†^ | 0.52 +/- 0.03 | 4.1 +/- 0.2 | 5.6 +/- 0.1 | 11.9 +/- 0.8 |
| DmFDL* | 0.78 +/- 0.17 | 5.6 +/- 0.4 | 6.6 +/- 0.6 | 11.1 +/- 0.9 |
| AmFDL^†^ | 0.26 +/- 0.09 | 1.2 +/- 0.1 | 1.3 +/- 0.1 | 4.7 +/- 0.8 |
| AmFDL* | 0.14 +/- 0.01 | 1.2 +/- 0.0 | 1.4 +/- 0.1 | 10.5 +/- 2.9 |
| CeHEX-2^†^ | 0.41+/- 0.08 | 21.7 +/- 5.5 | 14.8 +/- 1.3 | 122.9+/- 10.6 |
| CeHEX-3^†^ | 0.59 +/- 0.14 | 5.8 +/- 0.5 | 5.2 +/- 1.0 | 7.8 +/- 3.0 |
| CeHEX-4^†^ | 0.59 +/- 0.05 | 14.0 +/- 0.7 | 13.8 +/- 1.4 | 19.5 +/- 2.3 |

**Supplemental Table I. Enzymatic properties of recombinant purified hexosaminidases with pNP-β-GlcNAc and pNP-β-GalNAc as substrates.** Michaelis constant (*K*_m_), specific activity, turnover and ***k*_cat_**/*K*_m_ for pNP-β-GlcNAc (a) and pNP-β-GalNAc (b) are shown. Values represent averages +/- standard error. † enzymes produced in *P. pastoris*; * enzymes produced in *Trichoplusia ni* Hi5 cells.

| **DNA oligonucleotide** | **sequence** |
| --- | --- |
| DmFDL_EcoRI_fw | G**GAATTC**CAAGGAGTCACCAAGGCCCAG |
| DmFDL_NotI_rv | T**GCGGCCGC**TCAAATGCATTCGCCGGGATTCT |
| DmFDL_G400V_fw | TCCTTCTACTGTGTAGAGCCACCTTGCGGA |
| DmFDL_G400V_rv | TCCGCAAGGTGGCTCTACACAGTAGAAGGA |
| DmFDL_W506A_fw | TTACCGTTCAGGTCGCGGGTGGAAGTAC |
| DmFDL_W506A_rv | GTACTTCCACCCGCGACCTGAACGGTAA |
| AmFDL_ClaI_fw | AG**ATCGAT**AGCCCCGCCACTCGCCTCCCTTCAGC |
| AmFDL_NotI_rv | AT**GCGGCCGC**TCAGAGACATTTGCCCGGGTTC |
| AmFDL_StuI_fw* | CAA**AGGCCT**AGCCCCGCCACTCGCCTCCCT |
| CeHEX2_PstI_fw | AG**CTGCAG**CTTGACTCAATGCAGAAGGAACCA |
| CeHEX2_NotI_rv | AT**GCGGCCGC**TCATTTCTTGATTGGGAAATG |
| CeHEX2_SfiI_fw ^†^ | AAT**GGCCCAGCCGGCCA**CTTGACTCAATGCAGAAGGAA |
| CeHEX2_NotI_rv ^†^ | TAT**GCGGCCGC**TTTCTTGATTGGGAAATGC |
| CeHEX3_PstI_fw | AG**CTGCAG**GAAACCACGAAAAAGCCGTCTC |
| CeHEX3_NotI_rv | AT**GCGGCCGC**CTATGTACAAGTTTTTTCGCTTTTC |
| CeHEX4_PstI_fw | AG**CTGCAG**AATGATCGATCCAGTTATGCTGCT |
| CeHEX4_NotI_rv | AT**GCGGCCGC**TCAATTAGTAATCTCTGTTCGAG |

**Supplemental Table II.** Primers that were used in this study for the cloning of hexosaminidases into pPICzαA and pFastBac Dual vector backbones. Restriction sites are bold and underlined in the sequences. ^†^ primers for cloning into pPICzαA vector for C-terminal HIS tagged protein. * forward primer for cloning into pFastBac Dual containing the melittin signal sequence and N-terminal HIS tag downstream of the polyhedrin promoter sequence.

**Supplemental Figures**


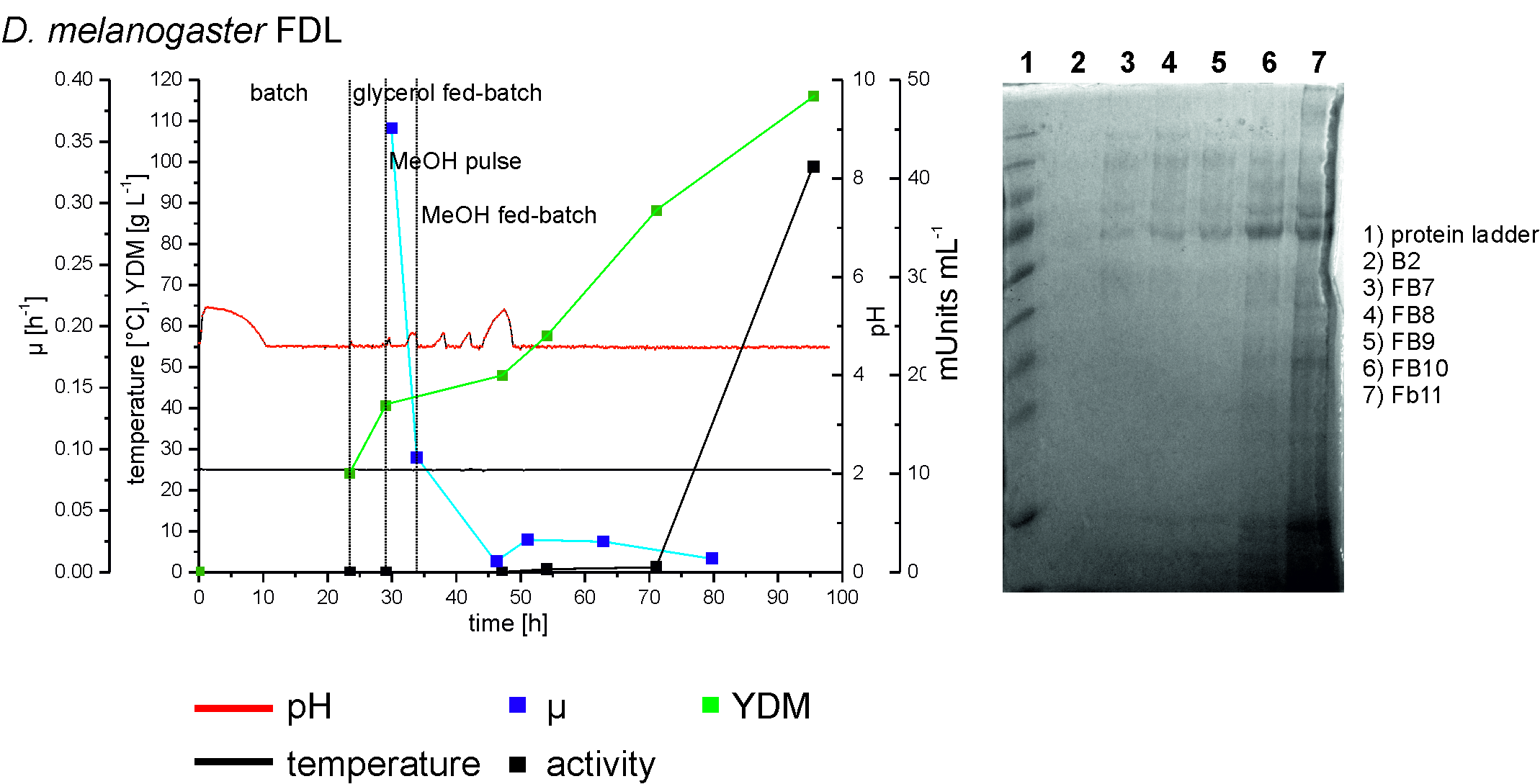


**Supplemental Figure 1. Bioreactor pilot expression of *Drosophila* FDL in *P. pastoris* X-33.** After a glycerol batch phase of approximately 28 hours, a glycerol fed-batch was initiated, followed by a methanol pulse and a constant methanol feed. After 95 hours a biomass yield of 115 YDM g L^-1^ and an activity in the supernatant of 40 mUnits mL^-1^ were achieved.

**
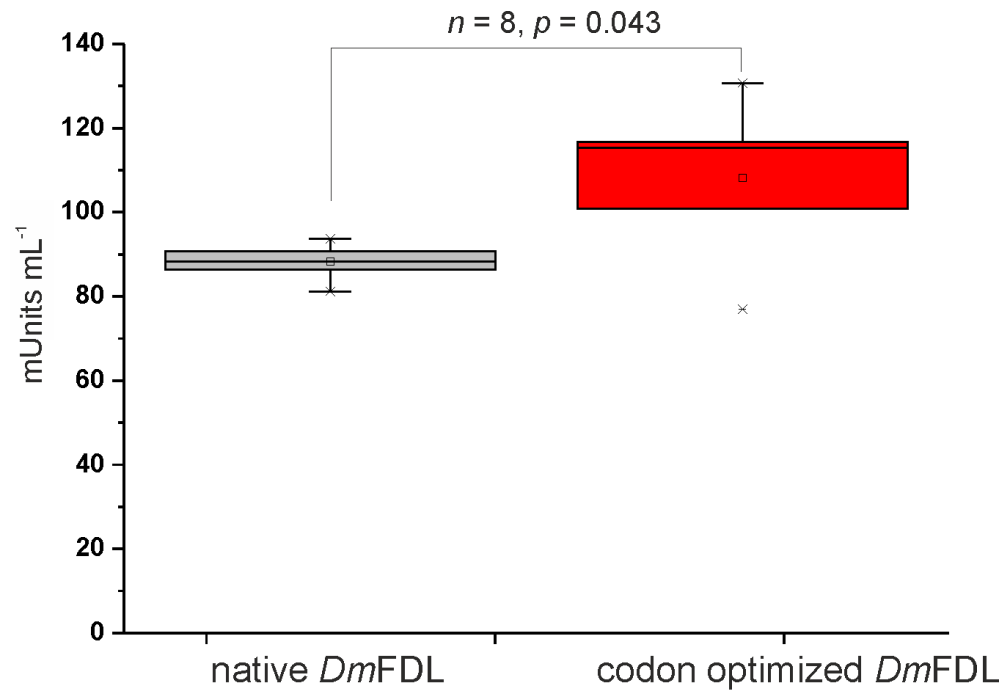
**

**Supplemental Figure 2. Influence of codon optimization on the yield (units per mL culture supernatant) of recombinant *Drosophila* FDL expressed in *P. pastoris* X-33.** Cultures were harvested after 48 hours of methanol induction at 25°C. Codon optimization increased the yield by approximately 25% (Student’s t-test, *p* = 0.043, number of biological replicates (*n*) = 8).





**Supplemental Figure 3. Product inhibition tests.** Standard pNP-β-GlcNAc assays were supplemented with GlcNAc (0 – 100 mM) or GalNAc (0 – 50 mM). For DmFDL and AmFDL data for recombinant proteins expressed in High Five cells are presented (recombinant products from *P. pastoris* showed the same result; data not shown). The *K*_i_ value for the GalNAc inhibition of the CeHEX4 was estimated by Dixon plot (data not shown). Values represent averages of triplicate measurements +/- standard error.

**
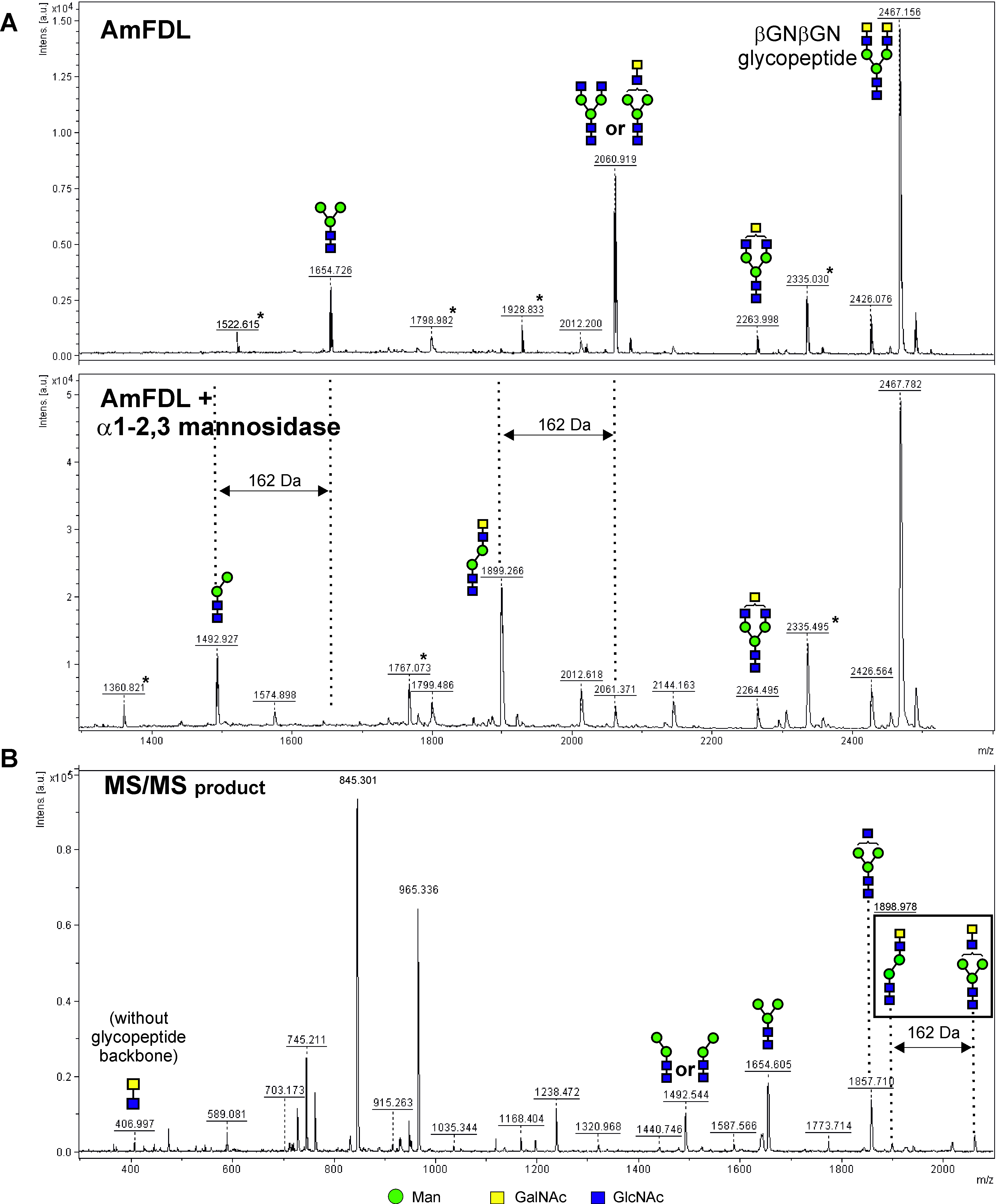
**

**Supplemental Figure 4. The recombinant AmFDL can process N-glycan substrates carrying terminal LacdiNAc.** Dabsylated glycopeptide carrying two terminal β-GalNAc residues was partially processed in presence of excess of the AmFDL enzyme produced in *P. pastoris* over an extended period of three days*.* The products were incubated in presence of α1,3-mannosidase, which facilitated removal of a terminal, α1,3-linked mannose residue indicating that a part of the AmFDL products are structures lacking one GlcNAc and one GalNAc residues, instead of two GalNAc residues (A). This result conforms with data obtained for the recombinant DmFDL enzyme as ions with m/z 407 and 1898 are also present in the in the MS/MS spectra of the relevant product (the structure with m/z 2060) (B). The glycans are depicted following the glycan nomenclature of the Consortium for Functional Glycomics (<http://www.functionalglycomics.org>). * The peaks derived from laser induced removal of the dabsyl group from the dabsylated glycopeptides.





**Supplemental Figure 5. Comparison of hexosaminidase activity in culture supernatants.** Enzyme activities of culture supernatants of *P. pastoris* wild type and a DmFDL expressing strain, as measured with pNP-β-GlcNAc as substrate, were compared to background hexosaminidase activities, which can be expected for 2 (2 d) and 4 days (4 d) old cultures of various insect cell lines (Sf9, *Spodoptera frugiperda* cells; Hi5, High Five *Trichoplusia ni* cells; TnAo38, *Trichoplusia ni* cells (Hashimoto, Y., Zhang, S., et al. 2012, Palmberger, D., Wilson, I.B.H., et al. 2012); S2, *Drosophila* Schneider 2 cells) and a HIS-tag purified non-hexosaminidase protein hemagglutinin from Hi5 cells (Krammer, F., Margine, I., et al. 2012). The dashed line indicates the hexosaminidase activity level that was achieved in *P. pastoris* cultures after 3 days of cultivation.





**Supplemental Figure 6. pH optima of purified DmFDL (A) and AmFDL (B) obtained from *P. pastoris* (squares) and High Five cells (circles).** Values represent averages +/- standard deviation of duplicate measurements.

**Supplemental References**

Hashimoto Y, Zhang S, Zhang S, Chen YR, Blissard GW. 2012. Correction: BTI-Tnao38, a new cell line derived from *Trichoplusia ni*, is permissive for AcMNPV infection and produces high levels of recombinant proteins. *BMC biotechnology*, 12:12.

Krammer F, Margine I, Tan GS, Pica N, Krause JC, Palese P. 2012. A carboxy-terminal trimerization domain stabilizes conformational epitopes on the stalk domain of soluble recombinant hemagglutinin substrates. *PLoS One*, 7:e43603.

Palmberger D, Wilson IBH, Berger I, Grabherr R, Rendic D. 2012. SweetBac: a new approach for the production of mammalianised glycoproteins in insect cells. *PLoS One*, 7:e34226.
